# Module-Selection Balance in the Evolution of Modular Organisms

**DOI:** 10.64898/2026.04.01.715873

**Published:** 2026-04-03

**Authors:** Mark Kim, Sarah M. Ardell, Sergey Kryazhimskiy

**Affiliations:** 1 Department of Ecology, Behavior and Evolution, University of California San Diego, La Jolla, CA 92093; 2 Department of Computational Biology, Cornell University, Ithaca, NY 14850; 3 Current address: Verdant Associates, Berkeley, CA 94707

## Abstract

The architecture of the genotype-phenotype-fitness map (GPFM) is a key determinant of evolutionary dynamics. One salient feature of biological GPFMs is variational modularity, where each mutation affects only a small subset of functional traits. Variational modularity may constrain the dynamics of trait evolution, but these constraints are not well understood. Here, we use several extensions of the Fisher’s geometric model with two functional traits to investigate these constrains. We find that on GPFMs with universal pleiotropy, populations evolve along the fitness gradient, which implies that the trait under stronger selection is optimized exponentially faster than the trait under weaker selection. In contrast, on modular GPFMs, populations approach a quasi-steady state that we term a “module-selection balance” where both traits improve at the same rate and their ratio remains constant. We demonstrate that the existence of a module-selection balance is robust with respect to the details of evolutionary dynamics and GPFMs themselves, as long as they are variationally modular. Our theory predicts that variationally modular organisms should exhibit stereotypical bi-phasic dynamics of genome evolution, especially in the strong clonal interference regime, and we find support for this prediction in metagenomic data from Lenski’s long-term evolution experiment in bacterium *Escherichia coli*. We propose that module-selection balance is an inherent feature of variationally modular GPFMs, which imposes an important constraint on long-term trait evolution.

## 1 Introduction

Heritable variation arises and is transmitted through generations at the genetic level, but natural selection acts on it only if this variation has a phenotypic manifestation. This duality implies that the efficiency of natural selection and the resulting dynamics of adaptation depend on the architecture of the genotype-phenotype-fitness map (GPFM). A salient feature of GPFMs of biological organisms is that they are modular, both in the functional and variational sense (Wagner et al., 2007). Functional modularity refers to the fact that different physiological functions are performed by distinct units, such as protein complexes, metabolic pathways or organs (Hartwell et al., 1999; Ravasz et al., 2002; Dudley et al., 2005; Bhattacharyya et al., 2006; Kreimer et al., 2008). Variational modularity refers to the property that most mutations affect only a small subset of key traits or functions (Wagner et al., 2008; Wang et al., 2010; Wagner and Zhang, 2011; Collet et al., 2018; Kinsler et al., 2020, 2024; Donyavi et al., 2025; Ghosh et al., 2026). Although many functional and variational modules coincide (e.g., each protein is encoded by a gene), this is not the case in general (e.g., different organs are not encoded by different parts of the genome). The ubiquity of modular GPFM architectures in biological systems raises two related but distinct questions (Wagner et al., 2007; Paaby and Rockman, 2013; Melo et al., 2016; Houle and Rossoni, 2022). Why and how do such architectures evolve? And how does modularity constrain the system’s evolutionary dynamics?

Much of the previous literature addressed the former question, with a particular focus on understanding the conditions in which modularity is favored by natural selection. Although the theory of evolutionary origins of modularity is still incomplete, the emerging consensus is that modularity can increase the system’s evolvability and thereby be favored by “second-order” natural selection (reviewed by Wagner et al., 2007; Melo et al., 2016; Houle and Rossoni, 2022). For example, *in silico* studies show that certain kinds of modularity can evolve if the organism’s fitness depends on multiple functions that share some sub-functions (Lipson et al., 2002; Kashtan and Alon, 2005; Kashtan et al., 2007; Sun and Deem, 2007; Parter et al., 2008; Kashtan et al., 2009a,b; He et al., 2009; Espinosa-Soto and Wagner, 2010; Tikhonov et al., 2020; Chebib and Guillaume, 2022). In addition, variational modularity can also evolve when it enables the organism to preferentially generate variation along phenotypic directions that are most favored by selection (Wagner, 1996; Welch and Waxman, 2003; Melo and Marroig, 2015; Tikhonov et al., 2020; do O and Whitlock, 2023).

The second question posed above has received less attention so far (but see Welch and Waxman, 2003; Schmiegelt and Krug, 2014; Park et al., 2015). Despite ample evidence that biological systems are modular, much of our understanding of trait evolution is based on the Fisher’s geometric model (FGM), which assumes “universal pleiotropy”, meaning that each mutation affects many if not all traits (Fisher, 1930; Hartl and Taubes, 1998; Orr, 2000; Martin and Lenormand, 2006, 2008; Sellis et al., 2011; Wagner and Zhang, 2011; Gordo and Campos, 2013; Weinreich and Knies, 2013; Tenaillon, 2014; Yamaguchi and Otto, 2020). Instead, the fact that organisms are variationally modular implies that most new mutations affect one or a few functional traits (Wagner et al., 2008; Wang et al., 2010; Wagner and Zhang, 2011; Collet et al., 2018; Kinsler et al., 2020, 2024; Donyavi et al., 2025; Ghosh et al., 2026), i.e., functional effects of new mutations are restricted or biased. Many recent theoretical and empirical studies have demonstrated that, in general, biases in the supply of mutations can affect evolutionary dynamics (Yampolsky and Stoltzfus, 2001; Stoltzfus and McCandlish, 2017; Storz et al., 2019; Gomez et al., 2020; Cano and Payne, 2020; Cano et al., 2022; Sane et al., 2023; Tuffaha et al., 2023; Gitschlag et al., 2023; Parveen et al., 2025). However, how biases imposed by variational modularity affect these dynamics remains unclear. Addressing this gap is the subject of this paper.

One constraint imposed by variational modularity has been recently observed in an experimental setting. Venkataram et al. (2020) evolved strains of *Escherichia coli* with defects in the translation machinery and found that natural selection sometimes failed to improve translation despite the availability of adaptive mutations in this module. Subsequent theoretical work showed that such “evolutionary stalling” of a trait can occur because of clonal interference between adaptive mutations in different modules (Gomez et al., 2020). Specifically, in rapidly adapting populations with limited recombination, clonal interference hinders the spread and fixation of all but the most beneficial mutations (Schiffels et al., 2011; Good et al., 2012; Lang et al., 2013; Gomez et al., 2020), so that only modules where such mutations are available can adapt, while other modules stall.

A difference in the rate of module improvement demonstrated by Venkataram et al. (2020) can over time lead to performance imbalances between traits, which in turn may have implications for the fitness of the organism in future environments. For example, microbes often adapt to nutrient-limiting conditions by acquiring mutations in nutrient transporters (Notley-McRobb and Ferenci, 1999; Gresham et al., 2008; Chou et al., 2009; Kvitek and Sherlock, 2011; Hong and Gresham, 2014; Bailey et al., 2014; Payen et al., 2014), even if some downstream functional modules, such as the translation machinery, may also be sub-optimal. However, these downstream modules may become limiting for fitness in future (e.g., nutrient-rich) environments. Thus, while there may be many combinations of traits that achieve the same fitness in the current environment, a modular architecture of the GPFM makes certain combinations more accessible than others, which has implications for the population’s future fitness. Importantly, these differences between trait combinations are invisible to selection in the current environment and are revealed only when the environment alters the phenotype-fitness map. Therefore, to understand how modularity shapes which traits improve, stall, or become imbalanced, we need to analyze not only changes in fitness but also how populations move in the trait space during adaptation.

These arguments motivate the central question of this study. We aim to understand how variational modularity shapes the evolutionary dynamics of traits as a population adapts to a constant environment. Our focus is not on which GPFM architectures are favored by selection but on how traits change through the course of adaptation on a given GPFM. To address this question, we consider the simplest model of the phenotype-fitness map where the fitness is a concave unimodal function of two underlying traits, with a single combination of trait values maximizing fitness, as in the classic Fisher’s geometric model (Fisher, 1930; Tenaillon, 2014) and related models (Wagner, 1989; Jones et al., 2003; Melo and Marroig, 2015). We compare and contrast the evolutionary dynamics that are induced in this trait space by two distinct genotype-phenotype maps, a fully modular one and one with universal pleiotropy. In our model of the fully modular GPFM, the two traits are encoded on different “chromosomes”, such that each mutation affects only one trait (Welch and Waxman, 2003). In contrast, on the GPFM with universal pleiotropy, both traits are encoded by the same genetic sequence, such that almost every mutation affects both traits. We use simulations of the Wright-Fisher model to obtrain evolutionary trajectories on these GPFMs with various population-genetic parameters. We derive analytical approximations where possible, which give us some insight into the general principles of how variational modularity constrains trait evolution. Lastly, we probe the robustness of our conclusions with respect to various extensions of our model and test them using metagenomic data from Lenski’s long-term evolution experiment.

## 2 Methods

### 2.1 Genotype-phenotype-fitness map models

We consider a phenotype-fitness map, which is based on the Fisher’s geometric model (FGM) with M=2 traits that contribute to fitness (Fisher, 1930; Tenaillon, 2014) (see Section 2.1.1 below). We construct the full genotype-phenotype-fitness map by coupling this phenotype-fitness model with four different genotype-phenotype map (GPM) models (see Sections 2.1.2-2.1.5). In all of our models, a genome with 2L bi-allelic loci, with alleles 0 and 1, is either implied or modeled explicitly, as specified below. In models where the genotypes are modeled explicitly, we consider two chromosomes, with each chromosome i containing $L_{i}$ loci, such that $L_{1}+L_{2}=2L$. We generally consider an additive genotype-phenotype map, except in the case of the nested FGM (see Section 2.1.5 below).

#### 2.1.1 Phenotype-fitness map

**Figure 1.**
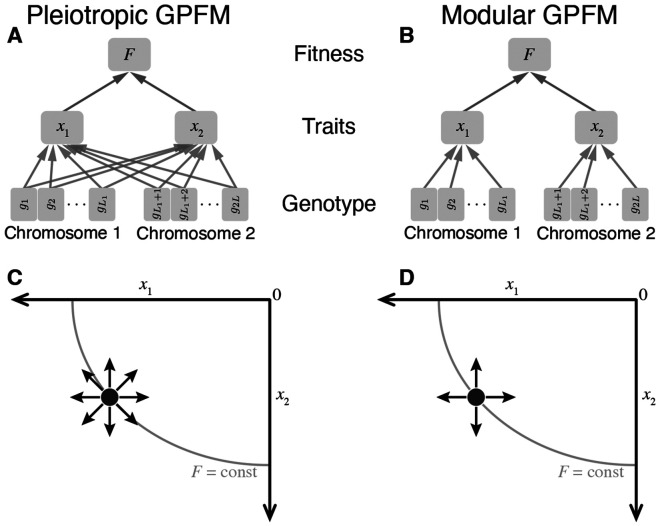
Schematic of the pleiotropic and modular genotype-phenotype-fitness maps (GPFMs). **A.** Pleiotropic GPFM. F is fitness; $x_{i}$ is the performance of the functional module i; $g_{\ell }$ is the allele (0 or 1) at site ℓ. Arrows indicate dependencies (see Section 2.1 for details). **B.** Modular GPFM. Notations are the same as in A. **C.** Mutational steps in the trait space of the pleiotropic GPFM. Black circle represents the current phenotype of an organism. A bit flip at site i moves the organism in the direction with angle $\theta_{\ell }$ relative to the $x_{1}$ axis (arrows). Angles $\theta_{\ell }$ are uniformly distributed over [0, 2π). Gray arc shows a fitness isocline. See Section 2.1.2 for details. **D.** Mutational steps in the trait space of the modular GPFM. Mutations 1 ↔ 0 on chromosome 1 only affect trait ($\theta_{\ell }=0$), mutations 1 ↔ 0 on chromosome 2 only affect trait $2(\theta_{\ell }=\pi ∕2)$. See Section 2.1.3 for details.

Our model of the phenotype-fitness map with two traits $x_{i}$, i=1,2 is illustrated in Figure 1A,B. We can think of each trait $x_{i}$ as representing the performance of a functional module i=1,2, relative to its optimum, so that $x_{i}=0$ corresponds to peak performance in the current environment. Thus, we refer to $x_{i}$ as either *trait* or *module performance*. We denote the position of an individual (or the position of the population mean) in the two-dimensional trait space by the trait vector $x=(x_{1},x_{2})$ (Figure 1C,D). If W is the Wrightian fitness, then we define the Malthusian fitness F=logW as a quadratic function of the trait vector x,

(1) $$F(x)=-\sum_{i=1}^{M}{\left(\frac{|x_{i}|}{a_{i}}\right)}^{2},$$

where the constants $a_{i}$ determine the contribution of trait i to organismal fitness and hence the strength of selection on the corresponding module, with smaller $a_{i}$ indicating stronger selection. Without loss of generality, we assume that $a_{2}<a_{1}$, i.e., trait 2 is under stronger selection than trait 1. We initialize both traits with negative values ($x_{i}<0$), so that adaptation corresponds to increases in $x_{i}$ toward zero.

#### 2.1.2 Pleiotropic GPM

Our main baseline genotype-phenotype map is a GPM with universal pleiotropy where each locus contributes to both traits, as shown in Figure 1A. We refer to the resulting GPFM as the “Pleiotropic GPFM”. Specifically, genotype $g=g_{1}g_{2}\cdots g_{2L}$, with $g_{\ell }\in \{0,1\}$, has trait values

(2) $$x_{1}(g)=\delta \sum_{\ell =1}^{2L}g_{\ell }\;\cos\;\theta_{\ell },\;\;x_{2}(g)=\delta \sum_{\ell =1}^{2L}g_{\ell }\;\sin\;\theta_{\ell },$$

where δ>0 is the magnitude of mutational effects and the angles $\theta_{\ell }$ are drawn independently from a uniform distribution on [0, 2π) (Figure 1C). We consider mutations that flip a bit at a single site ℓ. Flipping 1 → 0 at site ℓ changes the trait values by $\delta_{\ell 1}=-\delta \;\cos\;\theta_{\ell }$ and $\delta_{\ell 2}=-\delta \;\sin\;\theta_{\ell }$, giving a phenotypic displacement vector $\delta_{\ell }=(\delta_{\ell 2},\delta_{\ell 1})$. Flipping 0 → 1 yields the opposite effect $-\delta_{\ell }$.

In contrast to the canonical FGM, in our model, the number of mutational directions available to any genotype is finite and it varies across the trait space not only because of the geometry of the phenotype-fitness map but also because of the entropy of the underlying genotype space (Hwang et al., 2017). For example, the phenotypic optimum is encoded by a single genotype $g_{0}=00\cdots 0$. However, in large genomes (L≫1) and with uniformly distributed $\theta_{\ell }$, mutations are approximately isotropic at sufficiently populated trait values, and our pleiotropic GPM becomes asymptotically equivalent to the canonical FGM. Our analytical results are derived in this regime.

Specifically, in this approximation, the fitness effect of a mutation with angle θ that occurs in a genetic background with trait vector x is

(3) $$s(x,\theta )\;=\;F(x+\delta )-F(x)\;\;=\;-2\delta \left(\frac{x_{1}\;\cos\;\theta }{a_{1}^{2}}+\frac{x_{2}\;\sin\;\theta }{a_{2}^{2}}\right)-\delta^{2}\left(\frac{\cos^{2}\;\theta }{a_{1}^{2}}+\frac{\sin^{2}\;\theta }{a_{2}^{2}}\right)$$

(4) $$\approx \;-2\delta \left(\frac{x_{1}\;\cos\;\theta }{a_{1}^{2}}+\frac{x_{2}\;\sin\;\theta }{a_{2}^{2}}\right),$$

where the final approximation holds when $\delta \ll |x_{i}|$, i.e., when the population is sufficiently far from the optimum. In this case, a mutation is beneficial whenever

$$\theta_{0}(x)<\theta <\theta_{0}(x)+\pi ,\;\;\text{where},\;\;\theta_{0}(x)=\arctan\left(-\frac{x_{1}}{x_{2}}\frac{a_{1}^{2}}{a_{2}^{2}}\right).$$

#### 2.1.3 Modular GPM

Our main model is a GPM with variational modularity and we refer to the resulting GPFM simply as the “Modular GPFM” (Figure 1B). Here, loci on chromosome 1 contribute to trait 1 and loci on chromosome 2 contribute to trait 2, such that genotype $g=g_{1}g_{2}\cdots g_{2L}$ has trait values

(5) $$x_{1}(g)=-\delta \sum_{\ell =1}^{L_{1}}g_{\ell },\;\;x_{2}(g)=-\delta \sum_{\ell =L_{1}+1}^{2L}g_{\ell }.$$

As in the pleiotropic map, mutational effects have magnitude δ>0, both traits are additive, and the genotype 00 · · · 0 uniquely encodes the phenotypic optimum.

On this GPFM, any mutation 1 → 0 is beneficial. A beneficial mutation affecting module i has the selection coefficient

(6) $$s_{i}(x)=F(x+\delta_{i})-F(x)-{\left(\frac{\delta }{a_{i}}\right)}^{2}\left(-\frac{2x_{i}}{\delta }-1\right)$$

(7) $$\approx -\frac{2x_{i}\delta }{a_{i}^{2}}$$

as long as $\delta \ll |x_{i}|$, and the number of beneficial mutations available in module i is $b_{i}=|x_{i}|∕\delta$.

#### 2.1.4 Discordant-module GPM

In the modular GPM described above, functional and variational modules are concordant in the sense that each mutation affects only one functional module. To probe the robustness of our main results with respect to this assumption, we consider a generalization of the GPM model described above where functional and variational modules may be discordant. Specifically, we consider two chromosomes, such that all mutations on chromosome k=1, 2 affect the two functional traits in a ratio defined by a chromosome-specific angle $\theta_{k}\in [0,\pi ∕2]$. Without a loss of generality, we assume that $\theta_{1}<\theta_{2}$. As above, chromosome 1 contains $L_{1}$ binary loci $g_{1}g_{2}\cdots g_{L_{1}}\;(g_{\ell }\in \{0,1\})$, and chromosome 2 contains $L_{2}$ binary loci $g_{L_{1}+1}g_{L_{1}+2}\cdots g_{2L}$. We define the latent state of each chromosome k as

$$y_{1}(g)=-\delta \sum_{\ell =1}^{L_{1}}g_{\ell },\;\;y_{2}(g)=-\delta \sum_{\ell =L_{1}+1}^{2L}g_{\ell }.$$

Then the trait values are given by

(8) $$x_{1}=\sum_{k=1}^{2}y_{k}\;\cos\;\theta_{k},\;\;x_{2}=\sum_{k=1}^{2}y_{k}\;\sin\;\theta_{k}.$$

As on the GPFM with concordant modules, on this GPFM, any mutation 1 → 0 is beneficial. Thus, the number of beneficial mutations on chromosome k is $b_{k}=|y_{k}|∕\delta$, each such mutation changes the state of chromosome k by δ and has selection coefficient $s_{k}$ given by equations (3), (4) with $\theta_{k}$. We refer to this model as the “Discordant-module GPFM”.

#### 2.1.5 Nested Fisher’s geometric model

In the modular GPM described above, we assumed that both traits are additive and all mutations have exactly the same phenotypic effects δ. To probe the robustness of our main results with respect to these assumptions, we consider a “nested” Fisher’s geometric model, in which each trait is itself modeled with an FGM. Specifically, each functional trait $x_{i}$ is itself decomposed into $n_{i}$ more basal functional traits $y_{i}=(y_{i1},y_{i2},\ldots ,y_{in_{i}})$, with $x_{i}=-\frac{1}{2}\sum_{j=1}^{n_{i}}y_{ij}^{2}$. In this model, we maintain our original assumption that variational and functional modules are concordant, meaning that each mutation affects either trait $x_{1}$ or trait $x_{2}$. Since functional modules are by definition highly integrated, we assume that each mutation affecting trait $x_{i}$ is a vector $\delta_{i}$ in the $n_{i}$-dimensional space with a fixed magnitude $\|\delta_{i}\|=m$ and a random uniformly distributed direction. Thus, if a mutation with effect $\delta_{i}$ occurs in an organism with the trait vector $y_{i}$, the mutant’s trait vector is $y_{i}+\delta_{i}$, as in the canonical FGM (Tenaillon, 2014). The effect of such a mutation on the functional trait $x_{i}$ is $\delta_{i}$, with $\delta_{i}$ being approximately normally distributed with mean $-m^{2}∕2$ and standard deviation $m\sqrt{2|x_{i}|∕n_{i}}$ (Tenaillon, 2014). Consequently, both the fraction of beneficial mutations (those that increase $x_{i}$) and the distribution of their effect sizes $\delta_{i}$ are non-linear functions of the module’s current performance (Figure S4).

To make the nested FGM comparable to the modular GPFM, we set $m=\sqrt{2\delta }$, so that the mean magnitude of a mutation’s effect on $x_{i}$ equals $m^{2}∕2=\delta$, matching the fixed step size of the modular GPFM. With this calibration, the distribution of mutational effects on $x_{i}$ has mean −δ and standard deviation $2\sqrt{2\delta |x_{i}|∕n_{i}}$. We used $n_{1}=n_{2}=10$ underlying dimensions per module in all simulations; the consequences of unequal dimensionalities are explored in Figure S5. Implementation details are given in Section 2.4.

### 2.2 Models of evolutionary dynamics

The key population-genetic parameters that determine the evolutionary dynamics on our GPFMs are the mutation rate μ (per site per generation), the recombination rate ρ (per individual per generation) and the population size N. We also denote the total genome-wide mutation rate by U=2μL (per generation). The rate of beneficial mutations on a per site basis is $\mu_{b}$ (per generation), and the corresponding genome-wide rate of beneficial mutations is $U_{b}=2L\mu_{b}$ (per generation). In some models, we consider these rates on the chromosome rather than whole-genome basis, in which case they are denoted by $U_{k}$ or $U_{bk}$, k=1, 2. For simplicity, we only consider recombination between chromosomes (technically, reassortments) and ignore recombination within chromosomes.

We consider evolutionary dynamics in three regimes. (1) The *successive mutations* regime (also known as the “strong-selection weak-mutation”, or SSWM, regime) is defined by $NU_{b}\ll 1$, such that at any moment in time, at most one beneficial mutation is segregating in the population (Gillespie, 1991; Desai and Fisher, 2007; Kryazhimskiy et al., 2009; Good and Desai, 2015). In this regime, recombination plays no role. The *concurrent mutations* regime is defined by $NB_{b}>1$, such that multiple beneficial mutations segregate in the population at any moment (Desai and Fisher, 2007). In the concurrent mutations regime, we consider two extreme cases with respect to recombination. Regime (2) is when recombination is absent (ρ=0) and regime (3) is when chromosomes recombine freely (ρ=1).

The focus of our investigation is on the evolutionary trajectories in the trait space for an average adapting population. These average trajectories are described by the equation

(9) $$\frac{dx}{dt}=r(x),$$

where $r(x)=(r_{1}(x),r_{2}(x))$ is the vector of instantaneous rates of trait change at the trait-space location x. In particular, we are interested in how the balance between the two module performances changes during a bout of adaptation in a constant environment. We quantify this balance by the module performance ratio $R(t)=x_{2}(t)∕x_{1}(t)$, which measures the relative proximity of the two modules to their respective optima, such that R=1 indicates that both modules are equally close to their optima. It follows from equations (9) that R changes along the expected evolutionary trajectory according to equation

(10) $$\frac{dR}{dt}=\frac{r_{1}}{x_{1}}\left(\frac{r_{2}}{r_{1}}-R\right).$$

### 2.3 Analytical calculations

#### 2.3.1 Pleiotropic GPFM

For the pleiotropic GPFM, we carry out analytical calculations only in the successive mutations regime. Mutations with an angle in the infinitesimal interval (θ,θ, dθ) arise at rate NUdθ∕2π. Such a mutation has selection coefficient s(x,θ) given by equation (4). If the mutation is beneficial, it fixes with probability 2s(x,θ) and it is lost otherwise. A beneficial mutation improves trait 1 by δcosθ. Therefore, the expected rate of change in this trait is

(11) $$r_{1}=\int_{\theta_{0}}^{\theta_{0}+\pi }\frac{2NU}{2\pi }s(\theta )\delta \;\cos\;\theta \;d\theta =-\frac{NU\delta^{2}}{a_{1}^{2}}x_{1},\;\;\text{for}\;x_{1}<0,$$

and, analogously,

(12) $$r_{2}=-\frac{NU\delta^{2}}{a_{2}^{2}}x_{2},\;\;\text{for}\;x_{2}<0.$$

Substituting expressions (11), (12) into equation (9), we obtain equation (27) discussed in the Results section. Equation (27) implies that the population is approaching the optimum along the fitness gradient $\nabla F=(-2x_{1}∕a_{1}^{2},-2x_{2}∕a_{2}^{2})$. The solution of equation (27) is given by disp

(13) $$x_{i}(t)=x_{i0}e^{-\beta_{i}t},$$

where $x_{i0}$ is the initial value of trait i and $\beta_{i}=NU\delta^{2}∕a_{i}^{2}$. Eliminating t, we obtain the non-parametric equation (28) for the expected evolutionary trajectory in the trait space, which implies that the population approaches the optimum along the trait axis that corresponds to the module under weaker selection.

#### 2.3.2 Modular GPFM

##### Successive mutations regime.

On a modular GPFM, in the successive mutations regime, beneficial mutations in module i arise at rate $N\mu b_{i}=N\mu |x_{i}|∕\delta$ per generation and have selection coefficient $s_{i}(x)$ given by equation (7). The fraction $2s_{i}$ of them go to fixation, and each fixed mutation improves the respective trait by δ. Therefore, the expected rate of improvement of module i is $r_{i}=2N\mu |x_{i}|∕s_{i}$. Using expression (7) for $s_{i}$ in the regime $\delta \ll |x_{i}|$, we obtain

(14) $$r_{i}=\frac{4N\mu \delta x_{i}^{2}}{a_{i}^{2}}.$$

Substituting expression (14) into equation (9), we find that a typical population in the successive mutations regime moves along a trajectory defined by equations

(15) $$\frac{dx_{i}}{dt}=\alpha_{i}x_{i}^{2},\;\;i=1,2,\;\;\text{for}\;x_{i}<0,$$

where $\alpha_{i}=4N\mu \delta ∕a_{i}^{2}$. After eliminating t, we obtain

$$\frac{dx_{2}}{dx_{1}}=\frac{a_{1}^{2}}{a_{2}^{2}}\frac{x_{2}^{2}}{x_{1}^{2}},\;\;\text{for}\;x_{i}<0,$$

whose solution is given by equation (29) discussed in the Results section. Equation (15) can also be solved directly to yield

$$x_{i}=\frac{x_{i0}}{1-\alpha_{i}x_{i0},t},\;\;\text{for}\;x_{i0}<0.$$

#### Concurrent mutation regime with complete linkage.

To characterize how populations approach the module-selection balance line in the concurrent mutations regime with complete linkage, we define $v_{i}$, i=1, 2 to be the rate of fitness gains that is attributed to mutations improving trait i. We assume that the population is at the local beneficial mutation-selection balance (Desai and Fisher, 2007), which is determined by the current supplies of beneficial mutations in both modules $U_{bi}(x)=\mu b_{i}(x)=\mu |x_{i}|∕\delta$, their selection coefficients $s_{i}(x)$, which are given by equation (7), and the population size N.

We approximate $v_{i}$ (and hence $r_{i}=v_{i}\delta ∕s_{i}$) based on the results of Gomez et al. (2020). To this end, we first define $v_{i}^{\prime }$ to be the rate of adaptation of module i “in isolation”, i.e., the rate of adaptation of an organism with a single trait i. Desai and Fisher (2007) have shown that at the beneficial mutation-selection balance, $v_{i}^{\prime }\approx f_{\text{DF}}(s_{i},U_{bi},N)$ with

(16) $$f_{\text{DF}}\;(s,U_{b},N)=s^{2}\frac{2\;\log(Ns)-\log(s∕U_{b})}{\log^{2}(s∕U_{b})}.$$

Gomez et al. (2020) demonstrated that, if module j supports a much faster rate of fitness improvements in isolation than module i (mathematically, if $v_{i}^{\prime }\ll v_{j}^{\prime }$), then the rate of adaptation of module j in the two-module organism is approximately the same as its rate in isolation ($v_{j}\approx v_{j}^{\prime }$), whereas the slower module stalls $\left(v_{i}≲v_{i}^{\prime }\ll v_{j}^{\prime }\right)$. Moreover, they showed that the rates of trait evolution in a two-trait organism $v=(v_{1},v_{2})$ depend on the rates of evolution of the two isolated traits $v^{\prime }=(v_{1}^{\prime },v_{2}^{\prime })$ and are nearly independent of the specific combination of parameters $U_{bi}$ and $s_{i}$, i=1,2, that yield a particular $v^{\prime }$. We confirmed these findings using simulations (see Section 1.1 in Supplementary Information).

Given these results, we approximate the rates of improvement of trait i as

(17) ri=δsi×{0ifvi′vi¯′<1∕D,U~biU~bfDF(s~,U~b,N)if1∕D≤vi′vi¯′≤D,fDF(si,Ubi,N)ifvi′vi¯′>D,}

where $\bar{i}$ denotes the module other than i, $\tilde{s}=\sum_{i=1}^{2}s_{i}^{2}∕(s_{1}+s_{2})$ and ${\tilde{U}}_{bi}$ is a solution of equation

$$f_{\text{DF}}(\tilde{s},{\tilde{U}}_{bi},N)=f_{\text{DF}}(s_{i},U_{bi},N).$$

D denotes the ratio threshold, which we set to be D=100 although prediction quality is largely insensitive to this choice as long as D≫1 (Figure S3). The motivation for the approximation (17) as well as its validation are provided in Section 1.2 in Supplementary Information.

#### Concurrent mutation regime with free recombination.

We model evolution in this regime by assuming that the two modules evolve independently. In this case, sinces $s_{i}∕U_{bi}=2\delta^{2}∕(\mu a_{i}^{2})$, we have

(18) $$r_{i}=f_{\text{DF}}(s_{i},U_{bi},N)\frac{\delta }{s_{i}}=-\gamma_{i}x_{i}(2\;\log(-x_{i})+A_{i}),\;\;\text{for}\;x_{i}<0.$$

Here, $s_{i}$ and $f_{\text{DF}}$ are given by equations (7) and (16), respectively, $U_{bi}=\mu |x_{i}|∕\delta$, and

(19) $$\gamma_{i}=\frac{2\delta^{2}}{a_{i}^{2}\;\log^{2}\left(\frac{2\delta^{2}}{\mu a_{i}^{2}}\right)},\;\;A_{i}=\log\frac{2N^{2}\mu }{a_{i}^{2}}.$$

Substituting expression (18) into equation (9), we find that

(20) $$x_{i}(t)=-\text{exp}\left(-\frac{A_{i}}{2}+\left(\log(-x_{i0})+\frac{A_{i}}{2}\right)e^{-2\gamma_{i}t}\right),$$

which holds as long as each module adapts in the concurrent mutation regime, i.e., as long as the population stays sufficiently far from the phenotypic optimum, such that $2\;\log(-x_{i})+A_{i}>0$. Then, the expected trajectory in the trait space is given by equation

(21) $${\left[\frac{2\;\log(-x_{1})+A_{1}}{2\;\log(-x_{10})+A_{1}}\right]}^{\frac{1}{\gamma_{1}}}={\left[\frac{2\;\log(-x_{2})+A_{2}}{2\;\log(-x_{20})+A_{2}}\right]}^{\frac{1}{\gamma_{2}}}$$

and the module performance ratio is given by

(22) $$R(t)=\frac{a_{2}}{a_{1}}\;\text{exp}\left[\left(\log(-x_{20})+\frac{A_{2}}{2}\right)e^{-2\gamma_{2}t}-\left(\log(-x_{10})+\frac{A_{1}}{2}\right)e^{-2\gamma_{1}t}\right],$$

which implies that R eventually converges to $a_{2}∕a_{1}$. However, since $\gamma_{i}∼\delta^{2}$, this convergence is slow (compare to equation (15) where $a_{i}∼\delta$, and populations may transition to the successive mutations regime before they approach this equilibrium.

#### 2.3.3 Discordant-module GPFM

For the discordant-module GPFM, we carry out analytical calculations only in the successive mutations regime. Details are provided in Section 2 in Supplementary Information. Briefly, beneficial mutations on chromosome k arise at rate $N\mu b_{k}$, and fraction $2s\;(x,\theta_{k})$ of them fix, with s being given by equation (4). Each beneficial mutation reduces the chromosome state by δ. Then,

(23) $${\dot{y}}_{k}=2N\mu b_{k}s_{k}(x)\delta \approx 4N\mu \delta y_{k}\left(\frac{x_{1}\;\cos\;\theta_{k}}{a_{1}^{2}}+\frac{x_{2}\;\sin\;\theta_{k}}{a_{2}^{2}}\right).$$

It is then possible to show that the module performance ratio satisfies equation

(24) $$\dot{R}=-\alpha_{2}x_{1}P(R),$$

where $\alpha_{2}=4NU\delta ∕a_{2}^{2}$ as above and

$$P(R)=\frac{(\sin\;\theta_{2}-\sin\;\theta_{1})\;\cos\;\theta_{1}\;\cos\;\theta_{2}}{\sin\;(\theta_{2}-\theta_{1})}(P-{\bar{R}}_{1})\;(R-{\bar{R}}_{2})\;(R-{\bar{R}}_{3})\;,$$

with ${\bar{R}}_{i}=\tan\;\theta_{i}$ for i=1, 2 and

(25) $${\bar{R}}_{3}=\frac{a_{2}^{2}}{a_{1}^{2}}\frac{\cos\;\theta_{1}-\cos\;\theta_{2}}{\sin\;\theta_{2}-\sin\;\theta_{1}}.$$

In this model, R is confined to the interval [${\bar{R}}_{1}$, ${\bar{R}}_{2}$]. Given that and the fact that the sign of $\dot{R}$ is determined by the sign of P(R), it is possible to show that the module performance ratio R always stays positive and bounded and eventually converges either to ${\bar{R}}_{1}$ or to ${\bar{R}}_{3}$ (see Section 2 in Supplementary Information).

### 2.4 Simulations

We carried out two types of evolutionary simulations. For simulations in the successive mutations regime, we modeled the phenotypic state of the population as a Markov chain using the Gillespie algorithm (Gillespie, 1977). In all other cases, we simulated evolution using the Wright–Fisher model (Ewens, 2004).

Across all simulations, we set $a_{1}=1$, $a_{2}=1∕\sqrt{2}$, σ=2, $N=10^{4}$, δ=0.1, and $L_{1}=L_{2}=L=200$, i.e. both chromosomes have the same length and the full genome size is 2L=400. Mutations were parameterized by the per-locus mutation rate which we set $\mu =5\times 10^{-9}$ per locus per generation in the successive mutations regime and $\mu =10^{-5}$ per locus per generation in the concurrent mutations regime. The recombination rate was ρ=0 for simulations of the asexual regime and ρ=1 for simulations of the free recombination regime.

#### Numerical implementation of different GPFMs.

The four GPFMs differ in whether explicit genotypes are tracked and in how mutations are implemented, and these differences shape the simulation approach for each model. The pleiotropic GPFM is the only model in which each individual is characterized by an explicit binary genome of 2L=400 loci. In each simulation, each locus ℓ is assigned a fixed pleiotropic angle $\theta_{\ell }∼\text{Uniform}[0,2\pi )$, drawn once prior to simulation and shared across all replicates. Mutations at each locus arise at rate μ=U∕2L per individual per generation. A mutation at locus ℓ flips its allele (0 ↔ 1) and correspondingly displaces the phenotype by $\pm \delta_{\ell }$ as described in Section 2.1.2.

In the modular GPFM, we do not track genotypes explicitly because the number of loci on chromosome i that carry allele 1 contains all the information we need to carry out simulations. In particular, we do not need to know which specific loci carry allele 1 because the phenotypic effects at all loci are the same. The number of loci on chromosome i that carry allele 1 can be directly calculated from the trait value as $b_{i}=|x_{i}|∕\delta$. Thus, trait $x_{i}$ takes values on the discrete lattice $\left\{0,-\delta ,-2\delta ,\ldots ,-L_{i}\delta \right\}$. Beneficial mutations in module i arise at rate $\mu b_{i}$ per individual and shift the trait value $x_{i}$ by +δ; deleterious mutations arise at rate $\mu (L_{i}-b_{i})$ and shift the trait value $x_{i}$ by −δ. The initial trait values are discretized to the nearest δ-multiple and clamped to $x_{i}\leq 0$. Recombination is implemented by swapping the trait $x_{2}$ value between individuals in a mating pair.

The discordant-module GPFM is implemented analogously to the modular GPFM, except here we track the latent chromosome states $y=(y_{1},y_{2})$ because their value uniquely determines both the supplies of beneficial mutations on each chromosome and the functional trait values x.

In the nested FGM, mutations in module i arise at rate U∕2, and each such mutation shifts the trait $x_{i}$ by $\delta_{i}$ which is drawn from the normal distribution with mean $-m^{2}∕2$ and variance $2|x_{i}|m^{2}∕n_{i}$ (see Section 2.1). We set $m=\sqrt{2\delta }\approx 0.45$ and $n_{i}=10$. Recombination acts as in the modular GPFM, by swapping the trait $x_{2}$ between two individuals in a mating pair.

#### Simulations in the successive mutations regime.

In this regime, the population is assumed to remain monomorphic between fixation events, with transients to fixation treated as instantaneous. Since neutral and deleterious mutations do not contribute to state transitions in the successive mutations regime, we draw the waiting time τ until the appearance of the next beneficial mutation from an exponential distribution with rate $NU_{b}$, where $U_{b}$ is the genome-wide beneficial mutation rate, which is model-dependent (see below). We then determine the selection coefficient s of the arisen beneficial mutation, which is also model-dependent, and evaluate its probability of fixation using Kimura’s expression $P_{\text{fix}}(s)=(1-e^{-2s})∕(1-e^{-2Ns})$. If the mutation is lost, time advances by τ but the state of the population is unchanged; if it fixes, the state of the population is updated.

In the pleiotropic GPFM, to determine $U_{b}$, we evaluate the selection coefficients $s_{\ell }=s(x,\theta_{\ell })$ for all 2L loci according to expression (3). We form the set of beneficial loci $B=\{\ell :s_{\ell }>0\}$ as those with positive selection coefficients. The number of available beneficial mutations b is the size of this set, b=∣B∣, and $U_{b}=\mu b$ per individual per generation. Once a beneficial mutation arises, we draw a random locus ℓ from B which yields the selection coefficient $s_{\ell }$ of the arisen mutation.

In the modular and discordant-module GPFMs, the number of loci $b_{i}$ on chromosome i with available beneficial mutations is computed as described above. Then, $U_{b}=\mu (b_{1}+b_{2})$. We draw the chromosome i on which the beneficial mutation occurs with probability proportional to $b_{i}$. The mutant’s trait $x_{i}$ is then increased by +δ and its selection coefficient $s_{i}$ is evaluated according to equation (6).

In the nested FGM, we have $U_{b}=(U∕2)(P_{1}+P_{2})$, where $P_{i}=\mathrm{Pr}(\delta_{i}>0)$ is the probability that a random mutation on chromosome i is beneficial, computed analytically from the normal distribution specified above. The chromosome i on which the beneficial mutation occurs is chosen proportional to $P_{i}$. Then, the phenotypic effect $\delta_{i}$ is drawn from the normal distribution, conditional on $\delta_{i}>0$.

#### Wright-Fisher model simulations.

In the Wright-Fisher model simulations, each generation proceeds in three phases: mutation, recombination, and selection. Implementation of mutation and recombination events varies across GPFM models and is discussed below. After mutations and recombination events have occurred and the traits and fitness of all individual have been calculated, the selection step is applied uniformly across all models. The new generation is formed by sampling the number of descendants of each individual using multinomial sampling with N trials and probabilities proportional to individual Wrightian fitness $W=e^{F}$.

In the pleiotropic GPFM, the number of mutation events per generation is drawn from the Poisson distribution with mean NU. For each event, an individual who received the mutation and the locus at which the mutation occurs are drawn uniformly. Then, the allele at the specified locus is flipped and the phenotypes and fitness are updated accordingly. For ρ=1, all individuals are randomly paired and exchange their second chromosome containing loci $L_{1}+1,\ldots ,2L$.

In the modular and discordant-module GPFMs, the number of beneficial and deleterious mutations on chromosome k are drawn separately from the Poisson distribution with means $\sum_{j=1}^{N}\mu b_{kj}$ and $\sum_{j=1}^{N}\mu (L_{k}-b_{kj})$ respectively, where $b_{kj}$ is the supply of beneficial mutations on chromosome k in individual j and the sums run over all individuals. For each beneficial-mutation event, an individual is drawn randomly with probability proportional to $b_{kj}$, and its trait $x_{kj}$ (or chromosome state $y_{kj}$) is shifted by +δ. Similarly, for each deleterious-mutation event, an individual is randomly drawn with probability proportional to its $L_{k}-b_{kj}$ and its trait $x_{kj}$ (or chromosome state $y_{kj}$) is shifted by −δ. For ρ=1, all individuals are randomly paired, and trait values $x_{2}$ (or the latent chromosome states $y_{2}$) are swapped between mating partners.

In the nested FGM, the number of mutations per chromosome per generation is drawn from the Poisson distribution with mean NU∕2. An individual who receives the mutation is drawn uniformly and the trait increment $\delta_{i}$ is drawn from the normal distribution with mean $-m^{2}∕2$ and variance $2|x_{i}|m^{2}∕n_{i}$. For ρ=1, all individuals are randomly paired and $x_{2}$ is swapped between partners.

#### Initialization and termination.

Unless otherwise noted, all simulations are initialized at six locations in the trait space, each lying on the fitness contour $F_{0}=-1.39$ but with different module performance ratios $R_{0}=x_{20}∕x_{10}\in \{0.16,0.31,0.625,1.25,2.5,5\}$. We ran 250 replicate simulations per initial condition.

For the pleiotropic GPFM, we first draw the pleiotropic angle $\theta_{\ell }$ for each locus ℓ=1,…,2L independently from a uniform distribution on [0, 2π), shared across all initial conditions and realizations. Starting from the all-zero genome 00 · · · 0 (corresponding to the trait vector 0), we sequentially flip each locus 0 → 1 whenever doing so reduces the Euclidean distance to the target $x_{0}$. For the modular and nested FGMs, the initial population is monomorphic at $x_{0}$ in all regimes. For the discordant-module GPFM, the initial phenotype $x_{0}$ is first mapped onto latent chromosome state $y_{0}$ by solving linear equations (8), and the population is initialized monomorphically at $y_{0}$.

Simulations are terminated when the mean population fitness reaches $F_{f}=-0.01$, corresponding to Wrightian fitness $W_{f}=e^{F_{f}}=0.99$.

#### Visualization of the performance ratio dynamics.

**Figure 2.**
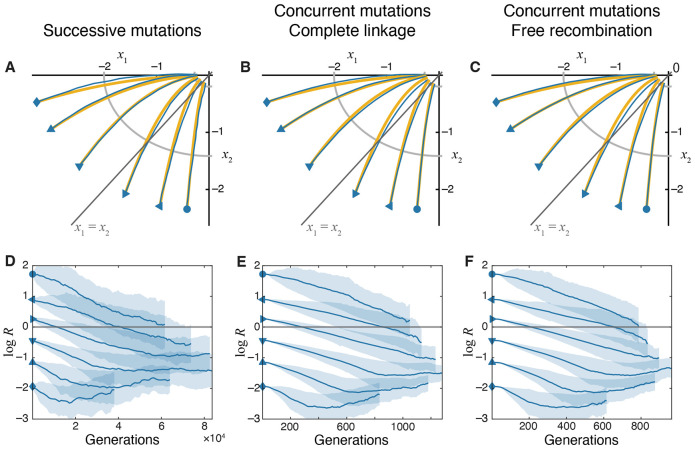
Evolutionary dynamics of traits on the pleiotropic GPFM. **A–C.** Trajectories in the trait space in different evolutionary regimes, as indicated. Populations are initialized at different locations in the trait space with the same fitness. Yellow lines show analytical predictions (equation (28)). Blue lines show simulated trajectories averaged over 250 replicates. Gray line indicates the diagonal $x_{1}=x_{2}$. **D–F**. Changes in the module performance ratio $R=x_{2}∕x_{1}$ over time in different evolutionary regimes. Solid lines show the mean computed and shaded bands show ±1 standard deviation of log R across replicates. Note that estimating log R in simulated populations becomes increasingly difficult when traits approach their optimal values, $|x_{i}|∼\delta$, and our method overestimates the true value of mean log R, causing these estimates to plateau or even increase at late times (see Section 2.4 in “Methods” for a longer discussion).

**Figure 3.**
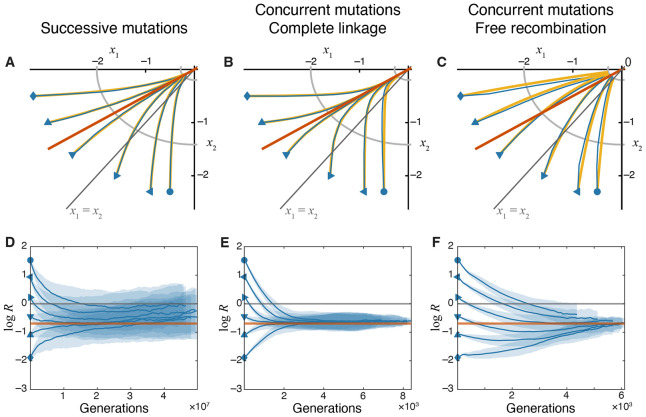
Evolutionary dynamics of traits on the modular GPFM. **A–C.** Trajectories in the trait space in different evolutionary regimes, as indicated. The orange line marks the module-selection balance defined by $s_{1}=s_{2}$ (equation (30)). Analytical predictions (yellow lines) are computed using equation (29) for panel A, equation (17) substituted into equation (9) and solved numerically for panel B, and equation (20) for panel C. Other notations are as in Figure 2. **D–F.** Changes in the module performance ratio $R=x_{2}∕x_{1}$ over time in different evolutionary regimes. The orange horizontal line marks the module performance ratio $\bar{R}=a_{2}^{2}∕a_{1}^{2}$ predicted at the module-selection balance. Other notations are as in Figure 2. Note that estimating log R in simulated populations becomes increasingly difficult when traits approach their optimal values ($|x_{i}|∼\delta$), and our method overestimates the true value of mean log R (see Section 2.4 in “Methods” for a longer discussion).

**Figure 4.**
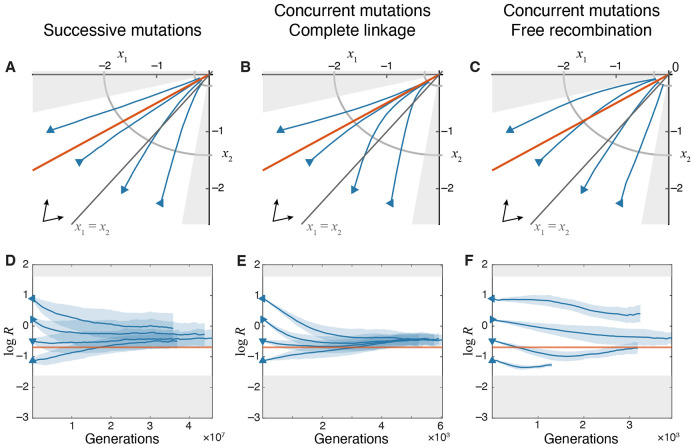
Evolutionary dynamics of traits on the discordant-module GPFM. **A–C.** Trajectories in the trait space in different evolutionary regimes, as indicated. The chromosome directions $\theta_{1}=\pi ∕16$ and $\theta_{2}=7\pi ∕16$ are indicated by arrows in the bottom left corner. The orange line marks the module-selection balance defined by $x_{2}∕x_{1}={\bar{R}}_{3}$ given by equation (25). Shaded regions indicate inaccessible areas of the trait space. Only those initial conditions are used for which $|y_{k}|\geq \delta$ for both k, i.e., at least one locus on each chromosome must carry the deleterious allele 1. Other notations are as in Figure 2. **D–F.** Changes in the module performance ratio $R=x_{2}∕x_{1}$ over time in different evolutionary regimes. The orange horizontal line marks the module performance ratio ${\bar{R}}_{3}$ predicted at the module-selection balance. Gray shaded areas indicate inaccessible values. Other notations are as in Figure 2. Note that estimating log R in simulated populations becomes increasingly difficult when traits approach their optimal values, $|x_{i}|∼\delta$, and our method overestimates the true value of mean log R (see Section 2.4 in “Methods” for a longer discussion).

**Figure 5.**
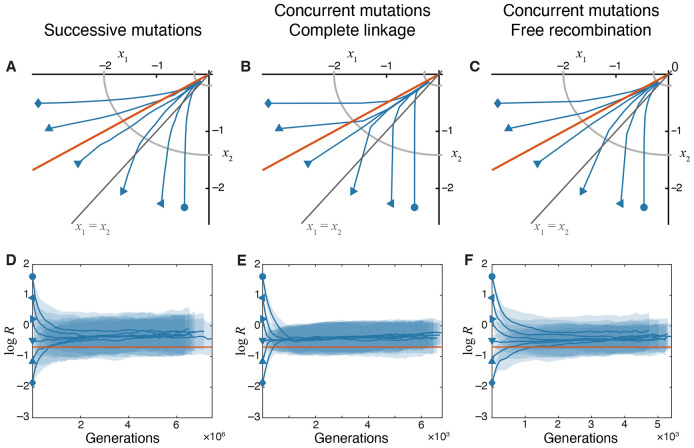
Evolutionary dynamics of traits on the nested FGM. **A–C.** Trajectories in the trait space in different evolutionary regimes, as indicated. The orange line marks the module-selection balance defined by $s_{1}=s_{2}$ (equation (30)). Other notations are as in Figure 2. **D–F.** Changes in the module performance ratio $R=x_{2}∕x_{1}$ over time in different evolutionary regimes. The orange horizontal line marks the module performance ratio $\bar{R}=a_{2}^{2}∕a_{1}^{2}$ predicted at the module-selection balance on the modular GPFM. Other notations are as in Figure 2. Note that estimating log R in simulated populations becomes increasingly difficult when traits approach their optimal values, $|x_{i}|∼\delta$, and our method overestimates the true value of mean log R (see Section 2.4 in “Methods” for a longer discussion).

When plotting log R against time (panels E–F in Figures 2-5 and S5), we exclude data points where either $x_{i}$ exceeds −δ to avoid logarithms of non-positive numbers or infinity. This procedure restricts the ensemble of populations that contribute to the estimate of mean log R and biases this estimate. In particular, since we chose $a_{2}<a_{1}$ for all our GPFMs, our populations tend to approach the optimal value of trait 2 faster than the optimal value of trait 1, which leads us to preferentially exclude populations with smaller values of R from our sample. Thus, our thresholding procedure biases the estimate of log R upwards when populations are close to at least one of their trait optima. Furthermore, since different simulations terminate at different times, estimates of mean log R become progressively noisier as time goes on. To avoid excessive noise, we display mean log R only at time points where at least 40 replicates still contribute.

### 2.5 Analysis of Lenski’s LTEE metagenomic data

We analyzed published metagenomic data from Lenski’s Long-Term Evolution Experiment (LTEE) obtained by Good et al. (2017) and available at https://github.com/benjaminhgood/LTEE-metagenomic. For each of the six non-mutator populations (Ara+1, Ara+2, Ara+4, Ara+5, Ara−5, Ara−6), we extracted all mutations assigned the “PASS” status in the annotated time course files. We excluded non-coding mutations because it is difficult to unambiguously assign them to specific genes. We retained a total of 1,464 genic mutations. We used the provided estimates of appearance times $t_{a}$ of each mutation, which were determined from a hidden Markov model analysis.

#### Sliding-window analysis.

To examine how the diversity of genes targeted by selection changes over the course of the experiment, we computed the effective number of gene targets in overlapping windows of the LTEE time course. Each window spans 10^4^ generations; windows are shifted by 2,500 generations. Mutations from all six populations are pooled within each window. For each window, we compute Simpson’s index $D=\sum_{i}p_{i}^{2}$, where $p_{i}$ is the fraction of mutations in gene i among all mutations observed in the current window, and report 1∕D as the effective number of genes under positive selection. To control for the unequal number of mutations per window, we subsampled 104 mutations per window without replacement, which is the minimum number of mutations across all qualifying windows (those containing at least 20 mutations prior to rarefaction). We computed 1∕D on each subsample and repeated this procedure 2 × 10^3^ times to obtain a bootstrap mean and 95% confidence interval.

#### Multi-hit gene analysis.

The PASS filter identifies variants that reached approximately 10% frequency in at least two consecutive sampled time points. As such, this set includes both driver and hitchhiker mutations. Since we do not expect the rate or the genomic distribution of hitchhiker mutations to change over time, we attribute all temporal changes in the distribution of observed mutations to drivers. Nevertheless, to test whether our results may be confounded by hitchikers, we also repeated the sliding-window analysis only for mutations found in multi-hit genes, i.e., genes with the number of independent mutations exceeding or equal to 2, 3, or 4. Sets of genes with increasingly high multiplicities are increasingly more enriched in targets of adaptation (Good et al., 2017), albeit at the expense of an increasing rate of false negatives, i.e., genes that are targets of adaptation but are not multi-hit. The results of this analysis are shown in Figure S6.

#### Genomic distribution.

To visualize how the genomic location of mutations shifts over the course of the experiment, we plotted the distribution of genomic positions of all genic PASS mutations separately for an early epoch ($t_{a}\leq 17,500$ generations) and a late epoch ($t_{a}>35,000$) generations. The late epoch was chosen as the shortest window that terminates at the final sampling time point and contains at least as many mutations as the early epoch.

### 2.6 Code availability

All the main simulations were performed in MATLAB (Mathworks, Inc.). LTEE analyses were performed in Python. Code is available at https://github.com/mkkim1894/ES_Project.

## 3 Results

### 3.1 On pleiotropic GPFM, populations adapt along the fitness gradient

To understand how variational modularity affects the evolutionary dynamics in the trait space, we first construct the null expectation for these dynamics on a GPFM with universal pleiotropy. In this model, an organism with the trait vector x has access to mutations with effectsδ=(δcosθ,δsinθ). For traits x represented by many genotypes, the angle θ is approximately uniformly distributed over [0, 2π) (see Section “Pleiotropic GPM” in “Methods” for details). When $\delta \ll |x_{i}|$, the selection coefficient of a mutation with angle θ is

(26) $$s\;(x,\theta )\approx -2\delta \left(\frac{x_{1}\;\cos\;\theta }{a_{1}^{2}}+\frac{x_{2}\;\sin\;\theta }{a_{2}^{2}}\right).$$

It is then straightforward to derive the expected rate of change in trait i in the successive mutations regime (Section “Pleiotropic GPM” in “Methods”),

(27) $$r_{i}=-\beta_{i}x_{i},\;\;\text{for}\;x_{i}<0,$$

with $\beta_{i}=NU\delta^{2}∕a_{i}^{2}>0$, which implies that the population approaches the optimum along the fitness gradient $\nabla F=(-2x_{1}∕a_{1}^{2},-2x_{2}∕a_{2}^{2})$. The gradient-ascent trajectory is given by

(28) $${\left(\frac{x_{1}}{x_{10}}\right)}^{a_{1}^{2}}={\left(\frac{x_{2}}{x_{20}}\right)}^{a_{2}^{2}},$$

which shows that, unless selection pressures on both modules are exactly identical ($a_{1}=a_{2}$), the module under stronger selection is optimized first, and the population approaches the fitness optimum by optimizing the remaining weakly-selected module (Figure 2A). This behavior can also be seen directly from the dynamics of the module performance ratio $R=x_{2}∕x_{1}$,

$$R(t)=R_{0}e^{-(\beta_{2}-\beta_{1})t}.$$

As long as $a_{1}\neq a_{2}$, the module performance ratio increases to infinity or decreases to zero, indicating that the relative proximity of the two modules to their respective optima diverges (Figure 2D).

Expression (28) for the expected evolutionary trajectories in the trait space was obtained for the successive mutations regime. To understand whether increasing the supply of mutations and adding recombination alters these trajectories, we carried out simulations in the concurrent mutations regime either with complete linkage between chromosomes (ρ=0) or when the chromosomes recombine freely (ρ=1, see Section 2.4 in “Methods” for details). We find that expression (28) remains reasonably accurate in these regimes, indicating that populations on the pleiotropic GPFM approach the fitness peak along trajectories of steepest ascent regardless of the population-genetic details (Figure 2C-F).

The fact that populations in the concurrent mutations regime adapt along the fitness gradient can be understood intuitively. When the supply of adaptive mutations is high, mutations that point in the direction of the gradient are likely to be present in the population. When linkage between chromosomes is complete, clones carrying these mutations tend to outcompete other clones. When recombination is obligate, it disrupts genotypes that provide the largest fitness benefits, but it also recreates them, so that the population still moves along the fitness gradient.

To summarize, on GPFMs with universal pleiotropy where the supply of mutations is unbiased, natural selection moves the populations along the fitness gradient, optimizing one trait at a faster rate than the other, which leads to their exponential divergence. This result provides a baseline expectation for our subsequent investigation of evolutionary dynamics in the presence of variational modularity.

### 3.2 On modular GPFM, populations evolve towards module-selection balance

To understand the effects of variational modularity on evolutionary dynamics, we first consider the simple Modular GPFM model (see Section 2.1.3). In this model, a beneficial mutation affecting module i has the selection coefficient $s_{i}\approx 2|x_{i}|\delta ∕a_{i}^{2}$ (see equation (7) in “Methods”), as long as $\delta \ll |x_{i}|$, and the number of beneficial mutations available in module i is $b_{i}=|x_{i}|∕\delta$.

Simulations show that variational modularity qualitatively changes how traits are improved by natural selection (Figure 3). Most importantly, populations with any parameters fail to evolve along the fitness gradient. As a result, the module under stronger selection is not necessarily optimized first. Furthermore, in contrast to the pleiotropic GFPM, recombination becomes an important factor on modular GPFMs (Figure 3C,F). To better understand these effects, we examine analytical approximations for the mean fitness trajectories on modular GPFMs.

#### Successive mutations regime.

In the successive mutations regimes, recombination can be ignored because the population is almost always monomorphic. In this regime, beneficial mutations in module i arise at rate $N\mu b_{i}=N\mu |x_{i}|∕\delta$ per generation. The fraction $2s_{i}$ of them go to fixation, and each fixed mutation improves the performance of the respective module by δ. Therefore, the expected rate of improvement of module i is $r_{i}=2N\mu |x_{i}|s_{i}$. Using expression (7) for $s_{i}$ in the regime $\delta \ll |x_{i}|$, we obtain $r_{i}=4N\mu \delta x_{i}^{2}∕a_{i}^{2}$ (see equation (14) in “Methods”), which shows that $r_{i}$ depends only on the current performance $x_{i}$ of module i but not on the performance of the other module, implying that equations (9) for the two traits decouple. Substituting expression (14) into equation (9), we find that a typical population whose initial trait vector is ($x_{10}$, $x_{20}$) evolves in the trait space along a hyperbolic trajectory

(29) $$\frac{a_{1}^{2}}{x_{10}}-\frac{a_{1}^{2}}{x_{1}}=\frac{a_{2}^{2}}{x_{20}}-\frac{a_{2}^{2}}{x_{2}}.$$

Therefore, all trajectories, irrespective of the initial condition, approach the optimum along the line

(30) $$\frac{x_{1}}{a_{1}^{2}}=\frac{x_{2}}{a_{2}^{2}}.$$

Equations (29), (30) show that, as the organism adapts, the ratio of module performances R approaches $\bar{R}=a_{2}^{2}∕a_{1}^{2}$ irrespective of the initial condition (Figure 3A,D).

This implies that, in contrast to a pleiotropic GPFM, on a modular GPFM, the strongly selected module eventually outperforms the weakly selected module only by a constant factor. We refer to this state of the population as the “module-selection balance”.

To further understand the biological significance of equation (30), recall that the fitness effect of a mutation at locus i is approximately given by equation (7). Therefore, equation (30) can be rewritten as

(31) $$s_{1}(x)=s_{2}(x),$$

which reveals that a typical population approaches the fitness peak along the line where selective benefits provided by adaptive mutations in both modules are identical (Figure 3A,D). In other words, the module-selection balance is achieved on the “equal fitness benefits” line in the trait space. Thus, on a modular GPFM, a population is expected to initially converge to the equal fitness benefits line and stay in this module-selection balance until it approaches the vicinity of the fitness peak where it will eventually reach the mutation-selection equilibrium (Hartl and Taubes, 1998; Sella, 2009; Nourmohammad et al., 2013).

#### Concurrent mutation regime with complete linkage.

Figures 3B,E show that populations with a large supply of adaptive mutations, i.e., those evolving in the concurrent mutations regime, also converge to the module-selection balance on the equal fitness benefits lines, at least when the module-encoding chromosomes are completely linked. In fact, it is possible to show that the equal fitness benefits line $L^{*}$ defined by equation (31) is a fixed point of equation (10) for the dynamics of the module performance ratio R. To demonstrate this fact, we first denote all quantities evaluated on line $L^{*}$ with an asterisk. On the equal fitness benefits line, beneficial mutations arise at rate $U_{b}^{*}(x)=U_{b1}^{*}(x)+U_{b2}^{*}(x)$, where $U_{bi}^{*}=\mu {|}_{i}^{*}|∕\delta$ is the rate of adaptive mutations in module i along $L^{*}$, and each provides a fitness benefit $s^{*}(x)$ relative to the parent. Then, assuming that the population is at the beneficial mutation-selection quasi-equilibrium (Desai and Fisher, 2007), its mean fitness increases at rate $v^{*}(x)=f_{\mathrm{DF}}(s^{*}(x),U_{bi}^{*}(x),N)$, with $f_{\mathrm{DF}}$ given by equation (16). Gomez et al. (2020) showed that, if the fitness benefits of mutations in two modules are identical (as is the case on $L^{*}$), the rate of fitness gains $v_{i}^{*}$ attributable to mutations in module i is proportional to $U_{bi}^{*}$. More precisely, $v_{i}^{*}=U_{bi}^{*}∕U_{b}^{*}v^{*}$, which implies that the rate of change in module performance is

(32) $$r_{i}^{*}=v_{i}^{*}\frac{\delta }{s^{*}}=\frac{\mu v^{*}}{U_{b}^{*}s^{*}}|x_{i}^{*}|\;.$$

Therefore, $r_{2}^{*}∕r_{1}^{*}=x_{2}^{*}∕x_{1}^{*}\equiv R^{*}$, which, according to equation equation (10), implies that dR∕dt=0 at any point on $L^{*}$. Thus, the equal fitness benefits line defined by equation (31) is indeed a fixed point for the dynamics of the module performance ratio R.

Populations adapting in the concurrent mutations regime approach the equal fitness benefits line along more straight trajectories than those in the successive mutations regime (compare Figures 3A and B). Indeed, when mutations in one module provide much bigger fitness gains than mutations in the other module, clonal interference effectively prevents fixation of the latter mutations and leads to evolutionary stalling of that module (Venkataram et al., 2020; Gomez et al., 2020). It is possible to derive analytical approximation for these trajectories using a heuristic approach based on the results of Gomez et al. (2020), which match our simulated results reasonably well (see equation (17) in “Methods”and Section 1.2 in Supplementary Information).

In summary, increasing the supply of adaptive mutations beyond the successive mutations regime does not qualitatively alter the evolutionary trajectories in the trait space on modular GPFMs, as long as genetic loci encoding both modules are linked. In this regime, populations reach a module-selection balance, in which module performances differ by a constant ratio, and maintain this balance while approaching the fitness peak.

#### Concurrent mutations regime with free recombination.

Figures 3C,F show that recombination between the loci encoding different modules significantly changes the evolutionary dynamics in the trait space. In particular, we see that populations approach the module-selection balance more slowly.

To understand these dynamics analytically, we model evolution in this regime under the assumption that the two modules evolve independently. In this case, we have (see equation (18)),

(40) $$r_{i}\approx -\gamma_{i}x_{i}\;(2\;\log(-x_{i})+A_{i})\;,\;\;\text{for}\;x_{i}<0,$$

where $\gamma_{i}$ and $A_{i}$ are positive constants given by expressions (19). Then the expected evolutionary trajectory in the trait space is described by equation (21), and the module performance ratio is given by equation (22), which shows that, as t→∞, the module performance ratio converges to $a_{2}∕a_{1}$ rather than $a_{2}^{2}∕a_{1}^{2}$ as in the absence of recombination, and the rate of approach is slow because it is proportional to $\delta^{2}$ compared to δ in the absence of recombination. However, it is important to note that the dynamical equations (18) that are used to derive expression (21) for the module performace ratio trajectory hold only when both modules are sufficiently far from their optima and evolve in the concurrent mutations regime (Desai and Fisher, 2007). As both module performances improve, their supplies of beneficial mutations diminish and they transition from the concurrent mutations regime to the successive mutations regime where they are expected to converge to the equal fitness benefits line given by equation (30).

This analysis shows that populations are indeed expected to eventually approach the same module-selection balance even in the presence of recombination, albeit at a slower rate.

### 3.3 Generalizations

So far, we found that populations approach a module-selection balance on the simplest modular GPFM. This model relies of several key assumptions. We assumed that (i) variational and functional modules are concordant, i.e., each “chromosome” encodes one trait, (ii) both traits are additive and (iii) all mutations have exactly the same phenotypic effects δ. Here, we relax each of these assumptions and ask whether module-selection balance still arises in these more general models. To relax assumption (i), we consider a model where the variational and functional modules are “discordant”, i.e., mutations on each chromosome contribute to both traits in a fixed ratio. Then, to relax assumptions (ii) and (iii), we consider the nested Fisher’s geometric model, in which each trait is itself modeled with an FGM. In the nested FGM, each population has access to mutations with a variety of phenotypic effects, and mutations exhibit epistasis at the level of each trait. We show that the module-selection balance is maintained in both cases.

#### Model with discordant variational and functional modules.

To assess whether the existence of a module-selection balance depends critically on the concordance between variational and functional modules, we consider a model with two chromosomes, where all mutations on chromosome k=1,2, 2 affect both functional traits in a ratio defined by a chromosome-specific angle $\theta_{k}\in [0,\pi ∕2]$, with $\theta_{1}\neq \theta_{2}$ (see Section 2.1.4 in “Methods”). It is easy to show that on this GPFM, trait combinations with ratios $R<{\bar{R}}_{1}$ and $R>{\bar{R}}_{2}$ are not admissible, with${\bar{R}}_{1}=\tan\;\theta_{1}$ and ${\bar{R}}_{2}=\tan\theta_{2}$ (see Section 2.3.3 in “Methods” and Section 2 in Supplementary Information for details). Furthermore, we show that in the successive mutations regime, the sign of the time derivative $\dot{R}$ is given by the sign of the third-degree polynomial with three roots ${\bar{R}}_{1}$, ${\bar{R}}_{2}$ and ${\bar{R}}_{3}$ given by equation (25), such that R always stays non-zero and bounded and eventually converges either to ${\bar{R}}_{1}$ or ${\bar{R}}_{3}$ (Figure 4). Thus, module-selection balance does not critically depend on the concordance between variational and functional modules.

#### Nested Fisher’s geometric model.

To investigate the robustness of our results with respect to the assumptions of additivity of functional traits and the absence of variation in the effects of mutations, we consider a hierarchical model where each functional trait $x_{i}$ is itself decomposed into $n_{i}$ more basic functional traits $y_{i}=(y_{i1},y_{i2},\ldots ,y_{in_{i}})$. Specifically, we let $x_{i}$ be a quadratic function of $y_{i},x_{i}=-\frac{1}{2}\sum_{j=1}^{n_{i}}y_{ij}^{2}$ (see Section 2.1.5 in “Methods”).

In this model, we maintain our original assumption that variational and functional modules are perfectly concordant, meaning that each mutation affects either trait $x_{1}$ or trait $x_{2}$. Since functional modules are by definition highly integrated, we assume that each mutation affecting trait $x_{i}$ is a vector $\delta_{i}$ in the $n_{i}$-dimensional space with a fixed magnitude $\|\delta_{i}\|=m$ and a random uniformly distributed direction, such that if a mutation with effect $\delta_{i}$ occurs in an organism with the trait vector $y_{i}$, the mutant’s trait vector is $y_{i}+\delta_{i}$, as in the canonical FGM (Tenaillon, 2014). Then, the effect of such a mutation on the functional trait $x_{i}$ is $\delta_{i}$, with $\delta_{i}$ being approximately normally distributed with a mean of $-m^{2}∕2$ and standard deviation $m\sqrt{2|x_{i}|∕n_{i}}$ (Tenaillon, 2014). Consequently, both the fraction of beneficial mutations (those that increase $x_{i}$) and the distribution of their effect sizes $\delta_{i}$ are non-linear functions of the module’s performance (Figure S4). We refer to this model as the nested FGM.

We simulated evolution in this model in the sequential as well as concurrent mutations regime in the presence and absence of recombination and found that populations still approach a module-selection balance (Figures 5 and S5). In particular, when both modules have the same underlying dimensionality $n_{i}$, the module performance ratio equilibrates remarkably close to the equal fitness benefits line (equation (30)) predicted in our simple modular GPFM model (Figure 5). When underlying module dimensionalities are different, module-selection balance is still achieved albeit at a different equilibrium performance ratio (Figure S5).

The results show that our main claim—the existence of a module-selection balance—is robust with respect to various implementations of variationally modular GPFM architectures. They suggest that it is variational modularity itself that forces natural selection to keep improving both functional traits at the same rate rather than nearly sequentially as in pleiotropic architectures.

### 3.4 Patterns of genome evolution in Lenski’s LTEE are consistent with the module-selection balance theory

We next asked whether real biological populations can be observed approaching a module-selection balance. According to our theory, one salient feature of adaptation on modular GPFMs is that, after an initial transient phase, both functional modules improve at the same rate, whereas on highly pleiotropic GPFMs, later stages of adaptation are characterized by improvements in a single (weakly selected) module. The difference in the shape of evolutionary trajectories on different GPFM architectures is most pronounced when populations are asexual and evolve in the concurrent mutations regime because such populations rapidly approach the module-selection balance (compare Figure 2 and Figure 3). An ideal test of this prediction would be based on observations of changes in functional traits of an asexual population evolving in a constant environment over thousands of generations. However, such data are difficult to obtain. Thus, we turn to published genomic data obtained from Lenski’s long-term evolution experiment (LTEE) (Good et al., 2017). In this experiment, 12 replicate populations of the bacterium *Escherichia coli* adapted over 60 thousand generations to a constant laboratory environment (Lenski et al., 1991; Lenski, 2017) and their whole metagenomes were sequenced by Good et al. (2017).

Although our theory does not explicitly describe the patterns of genome evolution, it is possible to obtain certain qualitative expectations. Specifically, our analytical calculations and simulations show that asexual populations evolving in the concurrent mutations regime on a variationally modular GPFM initially adapt by improving almost exclusively a single module (Figures 3B), i.e., they exhibit evolutionary stalling (Venkataram et al., 2020). If the number of traits contributing to fitness is larger than two, we would expect that perhaps several modules would be improving initially but the majority would be stalled. Regardless, driver mutations should be concentrated in relatively few genes comprising module or modules that are improving by natural selection. At later stages, we expect populations to approach the module-selection balance where multiple modules are being improved simultaneously, implying that driver mutations should be distributed widely across many genes. Once populations approach a module-selection balance, we expect the genomic distribution of driver mutations to remain constant and broad.

As mentioned above, we expect a largely opposite pattern of trait evolution on a highly pleiotropic GPFM (Figure 2B). However, the expectations at the genome level are less clear-cut for such GPFMs because trajectories depicted in Figure 2B could be consistent with multiple genomic distributions of driver mutations. Nevertheless, one can deduce genomic expectations under some reasonable assumptions. At one extreme, we can assume that mutations with various degrees of pleiotropy are distributed uniformly across the genome. In this case, we would expect a uniform distribution of driver mutations during the entire time course of evolution. Alternatively, we can assume that mutations with a specific degree of pleiotropy (i.e., particular value of angle θ in our model) are concentrated in certain genes. Since populations adapting on a pleiotropic GPFM approximately follow the fitness gradient, and since the direction of the gradient gradually changes as populations adapt, we would expect the genomic distribution of driver mutations to shift over time but not necessarily become broader or narrower.

This reasoning suggests that the main feature that distinguishes genome evolution on GPFMs with different architectures is how the breadth of the distribution of driver mutations along the genome changes over time. On pleiotropic GPFMs, we expect the breadth of this distribution to remain approximately constant, whereas on variationally modular GPFMs, we expect the breadth of this distribution to increase over time and then plateau. To test this prediction, we re-analyzed the metagenomic data from six non-mutator LTEE populations. Good et al. (2017) identified 1,464 genic mutations and their initial detection times across these populations (see Section 2.5 in “Methods”). They found that the initial detection times are non-randomly distributed, suggesting that the targets of natural selection shifted over time. In particular, they reported a significant enrichment of parallel mutations before ~17,500 generations.

**Figure 6.**
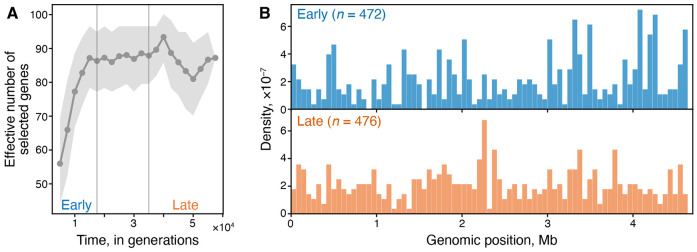
Changes in the genomic distribution of mutations in Lenski’s LTEE. **A.** Effective number of genes targeted by natural selection over time. Shaded region indicates 95% confidence intervals from 2 × 10^3^ bootstrap replicates (see Section 2.5 for methodological details). **B.** Genomic density of mutations that arose in the early (first 17,500 generations; *n* = 472 mutations) and in the late epoch (final 25,500 generations; *n* = 476 mutations). Data are from six non-mutator LTEE populations sequenced by Good et al. (2017).

To examine how the breadth of the genomic distribution of adaptive mutations changes over time, we compute the effective number of genes targeted by selection in 10^4^-generation sliding windows (see Section 2.5 in “Methods”). We find that this number rises sharply from ~55 at the beginning of the experiment to ~85 by roughly 17,500 generations, after which it remains approximately constant (Figure 6A). This pattern is also observed among mutations that occurred in multi-hit genes, which are strongly enriched for driver mutations (Figure S6). A direct visualization of the distribution of mutations along the genome reveals a clear clustering of early mutations at certain genomic loci and a more uniform distribution of late mutations (Figure 6B). This bi-phasic pattern is consistent with LTEE populations evolving on a variationally modular GPFM and reaching a module-selection balance after about ~ 17,500 generations.

## 4 Discussion

Here, we used several modifications and extensions of the classical Fisher’s geometric model to demonstrate how evolutionary dynamics in the trait space qualitatively change depending on the architecture of the underlying GPFM. On GPFMs with universal pleiotropy, an average population adapts by following the fitness gradient. As a result, the disparity in relative module performances grows over time without bound. Even though both modules improve, the module under stronger selection persistently pulls ahead, while the module under weaker selection continues to fall behind. In contrast, on modular GPFMs, an average population converges to what we term a “module-selection balance”—a state where the ratio of module performances remains constant over time—and then evolves towards the phenotypic optimum while maintaining this balance. The ratio of module performances at the module-selection balance as well as the rate of convergence to it are determined by the structure of the GPFM and the population genetic parameters, such as mutation and recombination rates. Convergence to this balance is fastest in asexual populations evolving in the concurrent mutations regime (Desai and Fisher, 2007), because clonal interference prevents fixation of all but the most beneficial mutations (Strelkowa and Lässig, 2012; Good et al., 2012). As a result, if one module pulls ahead, mutations improving it further become less abundant and less beneficial, which drastically decreases their chances of fixation and allows the lagging module to catch up and restore the balance. Recombination decouples the fates of mutations in different modules, which allows initial performance imbalances to persist longer. Yet, even in the absence of linkage, improvements in a module that pulls far ahead slow down, which prevents it from pulling ahead even further.

One key implication of these results is that modular organisms with initially arbitrarily different module performances are expected to converge towards a phenotypic state with the same module performance ratio. This is in contrast to highly pleiotropic GPFMs, on which evolutionary trajectories starting at different points in the trait space remain distinct. In a sense, modular organisms evolving in a constant environment “forget” their prior phenotypic state well before they reach the fitness optimum.

We discovered the module-selection balance in a relatively simple GPFM model, but found that this phenomenon is in fact preserved in more complex models. In particular, populations approach such balance on GPFMs where variational and functional modules are discordant (Figure 4) and, perhaps more surprisingly, on those generated by a nested FGM which allows for a non-linear dependence of the supply of adaptive mutations and their phenotypic effects on the distance to the optimum (Figure 5). These results lead us to the conjecture that the existence of a module-selection balance is a fundamental feature of long-term evolution on variationally modular GPFMs. Specifically, universal pleiotropy appears to be necessary for populations to follow the fitness gradient, which in turn appears to be required for the module performance ratio to either shrink or decline without bound, at least on smooth concave phenotype-fitness maps. In turn, variational modularity prevents populations from following the fitness gradient, which keeps the module performance ratio strictly positive and bounded. Evaluating this hypothesis and thereby more fully characterizing the types of GPFMs which admit a module-selection balance is an important open problem.

As discussed in the Introduction, our work was in part motivated by the empirical observation of evolutionary stalling (Venkataram et al., 2020) and the theoretical explanation behind it (Gomez et al., 2020), i.e., the fact that, in the presence of clonal interference, natural selection focuses on improving the lagging module while the leading one is stalled despite the availability of beneficial mutations in it. Our work places these previous results into a broader dynamical context. In particular, as suggested by Venkataram et al. (2020), we have shown that evolutionary stalling is a transient phase of adaptation that is supplanted by a module-selection balance where both modules improve at the same rate.

If biological organisms exhibit similar bi-phasic dynamics as observed in our simple model, we can make a non-trivial prediction about how the distribution of adaptive mutation along the genome should change during the course of long-term evolution in a constant environment. Specifically, if the underlying GPFM is variationally modular, natural selection is expected to initially be focused on improving one or a few modules whose performance is most strongly lagging behind. This would lead to a relatively narrow distribution of adaptive mutations along the genome. However, once the population approaches a module-selection balance, many modules should improve simultaneously, which should lead to a relatively broad distribution of adaptive mutations along the genome. Thus, we expect the genomic distribution of adaptive mutations to broaden over time on a variationally modular GFPM. In contrast, if the underlying GPFM is highly pleiotropic, we do not generically expect the breadth of this distribution to systematically change over time.

We tested this prediction in Lenski’s LTEE using metagenomic data obtained by Good et al. (2017) and found a strikingly bi-phasic change in the genomic distribution of putatively adaptive mutations, consistent with our module-selection balance theory (Figure 6). This dramatic shift in the width of the distribution of adaptive mutations occurs between 15 and 20 thousand generations. Coincidentally, fitness gains in LTEE populations dramatically slow down at approximately the same time, an observation that gave rise to the “two-epoch” hypothesis proposed earlier by Good and Desai (2015). It is possible that the first epoch, characterized by rapid fitness gains, corresponds to evolutionary stalling of many modules, whereas the second epoch, characterized by much slower fitness gains, corresponds to the module-selection balance.

The fact that the patterns in the metagenomic data from Lenski’s LTEE are consistent with our module-selection balance theory may appear trivial at first. After all, we know that most if not all organisms, including *E. coli*, have GPFMs that are at least to some extent variationally modular (Wagner and Zhang, 2011). Instead, the significance of this finding is threefold. First, it suggests that a module-selection balance—which was derived in a simple model with just two perfectly orthogonal modules—can in fact be achieved within ~ 10^4^ generations even on a potentially very complex GPFM with many imperfect modules. Second, if real populations indeed converge to a module-selection balance so rapidly, it suggests that a relatively short bout of adaptation in a constant environment is sufficient to erase most phenotypic differences that may be present between initial populations, much earlier than these populations would approach the fitness peak. Perhaps most importantly, module-selection balance implies that, even if the underlying trait space is high-dimensional, populations might approach fitness peaks along low-dimensional manifolds. This suggests that well-adapted organisms may admit particularly simple effective descriptions, consistent with several recent observations (e.g. Scott et al., 2010; Kinsler et al., 2020; Ardell et al., 2024).

## Supplementary Material

Supplement 1
