# Supplementary material for: Module-Selection Balance in the Evolution of Modular Organisms
Source: bioRxiv. 2026 Apr 3:2026.04.01.715873. Preprint. [Version 1] doi: 10.64898/2026.04.01.715873 (PMC13060315; doi:10.64898/2026.04.01.715873)
Supplement: Supplement 1 [file media-1.pdf]

Supplementary Information for

Module-Selection Balance in the Evolution of Modular Organisms

Mark Kim, Sarah M. Ardell, Sergey Kryazhimskiy*

*E-mail: skryazhi@ucsd.edu

1 Evolutionary dynamics on the modular GPFM in the concurrent mutations regime with complete linkage

1.1 Numerical confirmation of the results by Gomez et al. (2020)

Gomez et al. (2020) studied adaptation of an asexual population of an organism with two traits in the concurrent mutations regime. In their model, each trait k is characterized by its own selection coefficient s_k and beneficial mutation rate U_{bk}. Their central finding is that the rate of adaptation of each trait in a two-trait organism depends primarily on whether that trait has a faster or slower rate of adaptation in isolation (v'_k), and not on the specific combination of s_k and U_{bk} that produces v'_k. In particular, if one trait would adapt much faster than the other in isolation ($v'_j \gg v'_i$), then in a two-trait organism, this trait adapts at roughly the same rate as in isolation ($v_j \approx v'_j$), while the slower trait stalls ($v_i \lesssim v'_i \ll v'_j$). Conversely, when both traits would adapt at similar rates in isolation ($v'_1 \approx v'_2$), they continue to adapt at similar rates in a two-trait organism ($v_1 \approx v_2$). Here, we validate and extend these findings with our own Wright-Fisher simulations, across a range of parameter combinations.

Choice of parameters. We varied parameters s_1, U_{b1}, s_2, U_{b2} as follows. First, we chose the theoretically predicted rates of adaptation in isolation, v'_1 and v'_2, given by equation (16) in the main text, from 21 unique pairs spaced over a 6×6 logarithmic grid with $v'_2 \geq v'_1$ (Figure S1A), where the six values being 5×10^{-6}, 2.15×10^{-5}, 9.28×10^{-5}, 4.0×10^{-4}, 1.72×10^{-3}, 10^{-2} per generation. Then, for each target value of v'_i, we identified four distinct parameter combinations (s_i, U_{bi}) that yield v'_i by numerically solving equation $v'_i = f_{\text{DF}}(s_i, U_{bi}, N)$ with a fixed ratio s_i/U_{bi} which we varied over the range $\{10, 22, 46, 100\}$, so that $s_i/U_{bi} \gg 1$ to ensure that the Desai-Fisher approximation remains valid (Desai and Fisher, 2007). The resulting ranges of (s_i, U_{bi}) are shown in Figure S1E–F. This procedure generated $4 \times 4 \times 21 = 336$ total parameter combinations $(s_1, U_{b1}, s_2, U_{b2})$.

Steady-state Wright-Fisher simulations. For each parameter combination, we ran a Wright-Fisher simulation in which a population of N haploid asexual individuals carries two independently evolving traits, each subject to beneficial mutations that accumulate additively. At each generation, the fitness of each lineage is computed from its accumulated mutation counts (n_1, n_2) as $F = n_1 s_1 + n_2 s_2$. Offspring counts are then drawn by Poisson resampling with expected value N, with probabilities proportional to individual Wrightian fitness e^F. Mutations are assigned after the new generation is formed, as the total number of mutations across both traits is drawn binomially with rate $U_{b1} + U_{b2}$ per offspring, and each mutation is independently assigned to trait 1 with probability

$U_{b1}/(U_{b1}+U_{b2})$ and to trait 2 otherwise. We ran our simulations for a total of 1.5×10^4 generations with a burn-in of 10^4 generations to allow the fitness distribution within the population to reach a steady state. The gains in fitness attributable to each trait, v_1 and v_2, were estimated as the slope of the mean fitness component $\langle n_i s_i \rangle$ between generation $t_{\text{burnin}} = 10^4$ and the final generation. If fewer than two mutations in a module reached fixation during this interval, that module was classified as stalled; for visualization purposes, stalled modules were assigned the minimum detectable rate of 3×10^{-7} per generation (Figure S1B).

Results. Our simulations confirm three central findings of Gomez et al. (2020) that underpin the heuristic approximation developed in Section 1.2 in the main text.

1. Figures S1A,B show that the rates of adaptation $\boldsymbol{v} = (v_1, v_2)$ in a two-module organism depend primarily on the rates of adaptation in isolation $\boldsymbol{v}' = (v'_1, v'_2)$, and are largely insensitive to the specific values of s_i and U_{bi} that produce a given $\boldsymbol{v}$. Indeed, we see that the data points in Figure S1B that correspond to the same theoretical (v'_1, v'_2) pair but different underlying (s_i, U_{bi}) combinations cluster tightly together in the (v_1, v_2) plane.

2. Figure S1C shows that, when the two modules have very different rates in isolation ($v'_j/v'_i \gg 1$), the faster module improves in a two-module organism at a rate that is close to its rate in isolation ($v_j \approx v'_j$), while slower module contributes very little to adaptation ($v_i \ll v_j$), consistent with evolutionary stalling driven by clonal interference (Gomez et al., 2020; Venkataram et al., 2020).

3. Conversely, when both modules adapt at comparable rates in isolation ($v'_2/v'_1 \sim 1$), their rates of improvement in a two-module organism are also close to each other, $v_j/v_i \sim 1$ (Figure S1D). The heuristic approximation for v_i that we develop below operates in this regime.

1.2 Heuristic approximation for evolutionary dynamics

Based on the results of Gomez et al. (2020) discussed above, we approximate the evolutionary dynamics of a populations on the modular GPFM in the concurrent mutations regime with complete linkage using the following piecewise approach. We divide the phase space into three regions. Two regions describe the "stalling regime" where one module supports a much faster rate of adaptation in isolation than the other, i.e., $v'_j/v'_i \gg 1$. In this regime, we approximate the rates of module improvements as $r_i = 0$ for the slower module and $r_j = (\delta/s_j)\, v'_j = (\delta/s_j)\, f_{\text{DF}}(s_j, U_{bj}, N)$ for the faster module. The third region describes the "simultaneous improvement regime" where both modules support similar rates of adaptation in isolation, i.e., $v'_j/v'_i \sim 1$. In this regime, we apply the following heuristic approach for approximating $\boldsymbol{v}$. In general, v_i is an unknown function of five

parameters, $v_i = \phi(s_i, U_{bi}, s_j, U_{bj}, N)$. However, in the special case when the selection coefficients of mutations in the two modules are the same, $s_1 = s_2 = s$, Gomez et al. (2020) have shown that the rate of fitness gains v_i attributable to mutations in module i is given by

$$\phi(s, U_{bi}, s, U_{bj}, N) = \frac{U_{bi}}{U_b} f_{\mathrm{DF}}(s, U_b, N), \tag{S1}$$

where $U_{bi} = \mu|x_i|/\delta$ as before and $U_b = U_{b1} + U_{b2}$. Thus, we approximate an organism with access to two types of mutations with different selection coefficients s_1 and s_2 that arise with rates U_{b1} and U_{b2} by an organism with access to two types of mutations with the same selection coefficient $\tilde{s}$ that arise with rates $\tilde{U}_1$ and $\tilde{U}_2$. We then use the functional form (S1) as an ansatz for ϕ, i.e., we will look for effective parameters

$$\tilde{s} = \xi(s_1, U_{b1}, s_2, U_{b2}, N),$$
$$\tilde{U}_i = \eta(s_i, U_{bi}, s_j, U_{bj}, N),$$

such that

$$v_i = \phi(s_i, U_{bi}, s_j, U_{bj}, N) = \frac{\tilde{U}_i}{\tilde{U}} f_{\mathrm{DF}}(\tilde{s}, \tilde{U}_i, N), \tag{S2}$$

where $\tilde{U} = \tilde{U}_1 + \tilde{U}_2$.

Clearly, function ξ must be symmetric with respect to the relabelling of the two modules, i.e.,

$$\xi(s_1, U_{b1}, s_2, U_{b2}, N) = \xi(s_2, U_{b2}, s_1, U_{b1}, N). \tag{S3}$$

In addition, functions ξ, η must satisfy the basic constraints

$$\xi(s, U_{b1}, s, U_{b2}, N) = s, \tag{S4}$$
$$\eta(s, U_{bi}, s, U_{bj}, N) = U_{bi} \tag{S5}$$

that guarantee that the modules adapt at rates v_i^* given by the equation (S1) whenever $s_1 = s_2 = s$.

We first assume that we have already found a suitable value $\tilde{s}$ and discuss the choice of the function η. We return to the choice of the function ξ below. If $\tilde{s}$ is known, to set the effective mutation rates $\tilde{U}_i$, $i = 1, 2$, we use the observation by Gomez et al. (2020) that $\boldsymbol{v}$ depends primarily on $\boldsymbol{v}'$. Thus, we need to define $\tilde{U}_i$ so that it preserves the rate of adaptation v_i' of module i in isolation upon substituting s_i by $\tilde{s}$. This reasoning implies that we can define $\tilde{U}_i$ as a solution of equation

$$f_{\mathrm{DF}}(\tilde{s}, \tilde{U}_i, N) = f_{\mathrm{DF}}(s_i, U_{bi}, N). \tag{S6}$$

Note that, if $s_1 = s_2 = s$, then, according to the constraint (S4), $\tilde{s} = s$. Therefore, equation (S6) becomes

$$f_{\mathrm{DF}}(s, \tilde{U}_i, N) = f_{\mathrm{DF}}(s, U_{bi}, N),$$

which implies that the condition (S5) is satisfied.

We can rewrite equation (S6) as

$$v_i' \, x_i^2 + \tilde{s}^2 \, x_i - 2\tilde{s}^2 \log(N\tilde{s}) = 0,$$

where $x_i = \log(\tilde{s}/\tilde{U}_i)$, $v_i' = f_{\rm DF}\,(s_i, U_{bi}, N)$, and find that

$$x_i = \frac{-\tilde{s}^2 \pm \sqrt{\tilde{s}^4 + 8\tilde{s}^2 v_i' \log(N\tilde{s})}}{2v_i'},$$

which implies that one root is positive and the other is negative. For the Desai-Fisher approximation (equation (16) in the main text) to hold, we must have $\tilde{s}/\tilde{U}_i \gg 1$, i.e., x_i must be positive. Therefore, we set

$$\tilde{U}_i = \tilde{s} \exp\left[\frac{\tilde{s}^2 - \sqrt{\tilde{s}^4 + 8\tilde{s}^2 v_i' \log(N\tilde{s})}}{2v_i'}\right].$$

Now we turn to the choice of the function ξ. One simple class of functions that satisfy the constraints (S3), (S4) are the weighted averages

$$\tilde{s} = \xi(s_1, U_{b1}, s_2, U_{b2}, N) = \sum_{i=1}^{2} w_i \, s_i, \tag{S7}$$

with $w_1 + w_2 = 1$. In principle, the weights w_i can be any function of the population genetic parameters $s_1, U_{b1}, s_2, U_{b2}, N$. We tested three choices of these weights, (i) $w_i = 0.5$; (ii) $w_i = U_{bi}/U_b$; (iii) $w_i = s_i/(s_1 + s_2)$. To evaluate their accuracy, we plotted the rate of adaptation estimated empirically from our simulations against the theoretical values calculated using expression (S2) with $\tilde{s}$ given by equation (S7) and $\tilde{U}_i$ given by the solution of equation (S6). We found that all weight choices show similarly good accuracy (Figure S2) and we chose the weights $w_i = s_i/(s_1 + s_2)$, such that

$$\tilde{s} = \sum_{i=1}^{2} \frac{s_i^2}{s_1 + s_2}. \tag{S8}$$

Taking these results together, we approximate the rate of improvement of module i in a two-module organism as

$$r_i = \frac{\delta}{s_i} \times \begin{cases} 0 & \text{if } \frac{v_i'}{v_{\bar{i}}'} < 1/D, \\ \frac{\tilde{U}_i}{\tilde{U}} \, f_{\rm DF}(\tilde{s}, \tilde{U}, N) & \text{if } 1/D \le \frac{v_i'}{v_{\bar{i}}'} \le D, \\ f_{\rm DF}(s_i, U_{bi}, N) & \text{if } \frac{v_i'}{v_{\bar{i}}'} > D, \end{cases} \tag{S9}$$

where $\bar{i}$ denotes the module other than i and D denotes the ratio threshold. For all our subsequent calculations, we set the ratio threshold $D = 100$, but the specific choice of D is inconsequential as long as $D \gg 1$ (Figure S3). To obtain evolutionary trajectories in the trait space, we substitute expression (S9) into equation (9) in the main text and numerically solve it using MATLAB function ODE45.

2 Evolutionary dynamics on the discordant-module GPFM in the successive mutations regime

In the successive mutations regime, beneficial mutations on chromosome k arise at rate $N\mu b_k = N\mu|y_k|/\delta$, of which fraction $2s\left(\boldsymbol{x},\theta_k\right)$ go to fixation, with s being given by equation (4) in the main text. Each such fixed mutation reduces the chromosome state y_k by δ. Then,

$$\dot{y}_k = 2N\mu y_k s_k\left(\boldsymbol{x}\right) \approx \alpha\, y_k \left(\frac{x_1\cos\theta_k}{a_1^2} + \frac{x_2\sin\theta_k}{a_2^2}\right) \tag{S10}$$

with $\alpha = 4NU\delta$.

To simplify notations, we define $C_i = a_1^{-1}\cos\theta_i$, $S_i = a_2^{-1}\sin\theta_i$, $\xi_i = x_i/a_i$, and $r = \xi_2/\xi_1 = R\,a_1/a_2$. Then, equations (S10) become

$$\dot{y}_k = \alpha\, y_k\left(C_k\xi_1 + S_k\xi_2\right), \tag{S11}$$

and we have

$$\xi_1 = C_1 y_1 + C_2 y_2, \tag{S12}$$

$$\xi_2 = S_1 y_1 + S_2 y_2, \tag{S13}$$

from which we find that

$$y_1 = \frac{S_2\xi_1 - C_2\xi_2}{C_1S_2 - C_2S_1}, \tag{S14}$$

$$y_2 = \frac{-S_1\xi_1 + C_1\xi_2}{C_1S_2 - C_2S_1}. \tag{S15}$$

Since $\theta_2 > \theta_1$, it follows that $C_1S_2 - C_2S_1 = \left(a_1a_2\right)^{-1}\sin\left(\theta_2-\theta_1\right) > 0$, i.e., the denominator in equations (S14),(S15) is strictly positive, which means that the sign of y_k is determined by the respective numerators. The fact that $y_k \leq 0$ by definition, indicates that

$$\bar{r}_1 \leq r \leq \bar{r}_2, \tag{S16}$$

where we denoted $\bar{r}_1 = S_1/C_1$, $\bar{r}_2 = S_2/C_2$.

It follows from equations (S11)–(S15) that

$$\dot{\xi}_1 = \tilde{\alpha}\left[\tilde{A}_1\xi_1^2 + \tilde{B}_1\xi_1\xi_2 + \tilde{C}_1\xi_2^2\right], \tag{S17}$$

$$\dot{\xi}_2 = \tilde{\alpha}\left[\tilde{A}_2\xi_1^2 + \tilde{B}_2\xi_1\xi_2 + \tilde{C}_2\xi_2^2\right], \tag{S18}$$

where

$$\tilde{\alpha} = \alpha\frac{S_2 - S_1}{C_1S_2 - C_2S_1},$$

and

$$\tilde{A}_1 = \frac{C_1^2 S_2 - C_2^2 S_1}{S_2 - S_1}, \qquad \tilde{A}_2 = -S_1 S_2 \frac{C_2 - C_1}{S_2 - S_1},$$

$$\tilde{B}_1 = \frac{C_2 - C_1}{S_2 - S_1} \left(C_1 C_2 - S_1 S_2\right), \qquad \tilde{B}_2 = C_1 C_2 - S_1 S_2,$$

$$\tilde{C}_1 = C_1 C_2, \qquad \tilde{C}_2 = \frac{C_1 S_2^2 - C_2 S_1^2}{S_2 - S_1}.$$

Therefore,

$$\dot{r} = -\tilde{\alpha}\xi_1 P(r), \tag{S19}$$

where

$$P(r) = \tilde{C}_1 r^3 + \left(\tilde{B}_1 - \tilde{C}_2\right) r^2 + \left(\tilde{A}_1 - \tilde{B}_2\right) r - \tilde{A}_2$$

is a third-degree polynomial in r. Using the fact that

$$\tilde{B}_1 - \tilde{C}_2 = -\frac{C_1 - C_2}{S_2 - S_1} C_1 C_2 - \left(C_1 S_2 + C_2 S_1\right),$$
$$\tilde{A}_1 - \tilde{B}_2 = \frac{C_1 - C_2}{S_2 - S_1} \left(C_1 S_2 + C_2 S_1\right) + S_1 S_2,$$

it is straightforward to show that $P(r)$ factorizes as

$$P(r) = C_1 C_2 \left(r - \frac{S_1}{C_1}\right) \left(r - \frac{S_2}{C_2}\right) \left(r - \frac{C_1 - C_2}{S_2 - S_1}\right),$$

which means that the dynamics of r has three potential fixed points, $\bar{r}_1 = S_1/C_1$, $\bar{r}_2 = S_2/C_2$ and $\bar{r}_3 = \left(C_1 - C_2\right)/\left(S_2 - S_1\right)$, all of which are positive. Furthermore, since $\tilde{\alpha} > 0$ and $\xi_1 < 0$, equation (S19) shows that the sign of $\dot{r}$ (and hence the sign of $\dot{R}$) is determined by the sign of $P(r)$.

As noted above, r is confined to the interval $[\bar{r}_1, \bar{r}_2]$ (see equation (S16)). Thus, to understand the dynamics of r, we need to determine the location of the third root $\bar{r}_3$ relative to $\bar{r}_1$ and $\bar{r}_2$. First, we find that

$$\bar{r}_2 - \bar{r}_3 = \frac{(a_1^2 - a_2^2)\sin\theta_2\left(\sin\theta_2 - \sin\theta_1\right) + a_2^2\left(1 - \cos\left(\theta_2 - \theta_1\right)\right)}{a_1 a_2 \cos\theta_2\left(\sin\theta_2 - \sin\theta_1\right)} > 0$$

because $a_1 > a_2$, and $\theta_2 > \theta_1$. In contrast,

$$\bar{r}_3 - \bar{r}_1 = \frac{(a_1^2 - a_2^2)\sin\theta_1\left(\sin\theta_1 - \sin\theta_2\right) + a_2^2\left(1 - \cos\left(\theta_2 - \theta_1\right)\right)}{a_1 a_2 \cos\theta_2\left(\sin\theta_2 - \sin\theta_1\right)}$$

can be either positive or negative depending on the values of θ_1 and θ_2. For example, when $\theta_1 = 0$ and $\theta_2 = \pi/2$, we have $\bar{r}_3 > \bar{r}_1$, but when $\theta_2 = \theta_1 + \Delta\theta_1$ where $\Delta\theta \ll 1$, we have $\sin\theta_1 - \sin\theta_2 = -\cos\theta_1 (\Delta\theta) + o(\Delta\theta) < 0$ and $1 - \cos(\theta_2 - \theta_1) = o(\Delta\theta)$, which implies that $\bar{r}_3 < \bar{r}_1$.

This analysis shows that the roots of the polynomial $P(r)$—whose sign determines the direction of change of the module performance ratio R—can be arranged as $\bar{r}_3 < \bar{r}_1 < \bar{r}_2$ or $\bar{r}_1 < \bar{r}_3 < \bar{r}_2$, depending on the angles θ_1 and θ_2. If $\bar{r}_3 < \bar{r}_1 < \bar{r}_2$, then $P(r) < 0$ whenever $r \in (\bar{r}_1, \bar{r}_2)$ and r converges to $\bar{r}_1$. If $\bar{r}_1 < \bar{r}_3 < \bar{r}_2$, then $P(r) > 0$ on the interval $(\bar{r}_1, \bar{r}_3)$ and $P(r) < 0$ on the interval $(\bar{r}_3, \bar{r}_2)$, such that r converges to $\bar{r}_3$. Correspondingly, the module performance ratio R converges either to $\bar{R}_1 = a_2/a_1\bar{r}_1 = \tan\theta_1$ or to $\bar{R}_3 = a_2/a_1\bar{r}_3 = \frac{a_2^2}{a_1^2}\frac{\cos\theta_1 - \cos\theta_2}{\sin\theta_2 - \sin\theta_1}$ in these respective parameter regimes. Importantly, r (and hence R) always stays bounded, even if $\bar{r}_2 = \infty$ (which occurs when $\theta_2 = \pi/2$). Furthermore, r (and hence R) never decays to zero because even if $\bar{r}_1 = 0$ (which occurs when $\theta_1 = 0$), r converges to $\bar{r}_3 > 0$.

3 Supplementary Figures

Figure S1. Numerical validation of results by Gomez et al. (2020) (next page). A. Empirically estimated rates of adaptation in isolation v_i' (solid circles) and the respective theoretical values based on equation (16) in the main text (open circles). Each data point corresponds to a unique combination of parameters s_1, U_{b1}, s_2, U_{b2}. Population size is $N = 10^4$. Points are color-coded by the ratio v_2'/v_1': black $= 1$; blue $= 10$; dark green $= 10^2$; purple $= 10^3$; orange $\geq 10^4$. Dashed line is the diagonal. **B.** Empirically estimated rates of adaptation in a two-module organism v_i. Colors correspond to panel A. If fewer than two mutations reached fixation, the module is classified as stalled and its rate is set to the minimum detectable value 3×10^{-7} per generation (vertical dashed line). **C.** Two-module rate v_i (y-axis) plotted against the corresponding rate in isolation v_i' (x-axis) in the "stalling regime" where $v_2'/v_1' > 100$. Crosses ($\times$) denote v_1; filled circles denote v_2. Colors correspond to panel A. The dashed line represents $v_i = v_i'$. **D.** Same as C but in the "simultaneous improvement regime" where $v_2'/v_1' \leq 100$. **E–F.** Ranges of (s_i, U_{bi}) corresponding to the parameter grid in panels A–D. For each theoretical pair (v_1', v_2'), four values of $a_i \in \{10, 22, 46, 100\}$ are used to back-calculate (s_i, U_{bi}) per module, yielding $4 \times 4 = 16$ unique parameter combinations per grid point. Colors correspond to panel A.

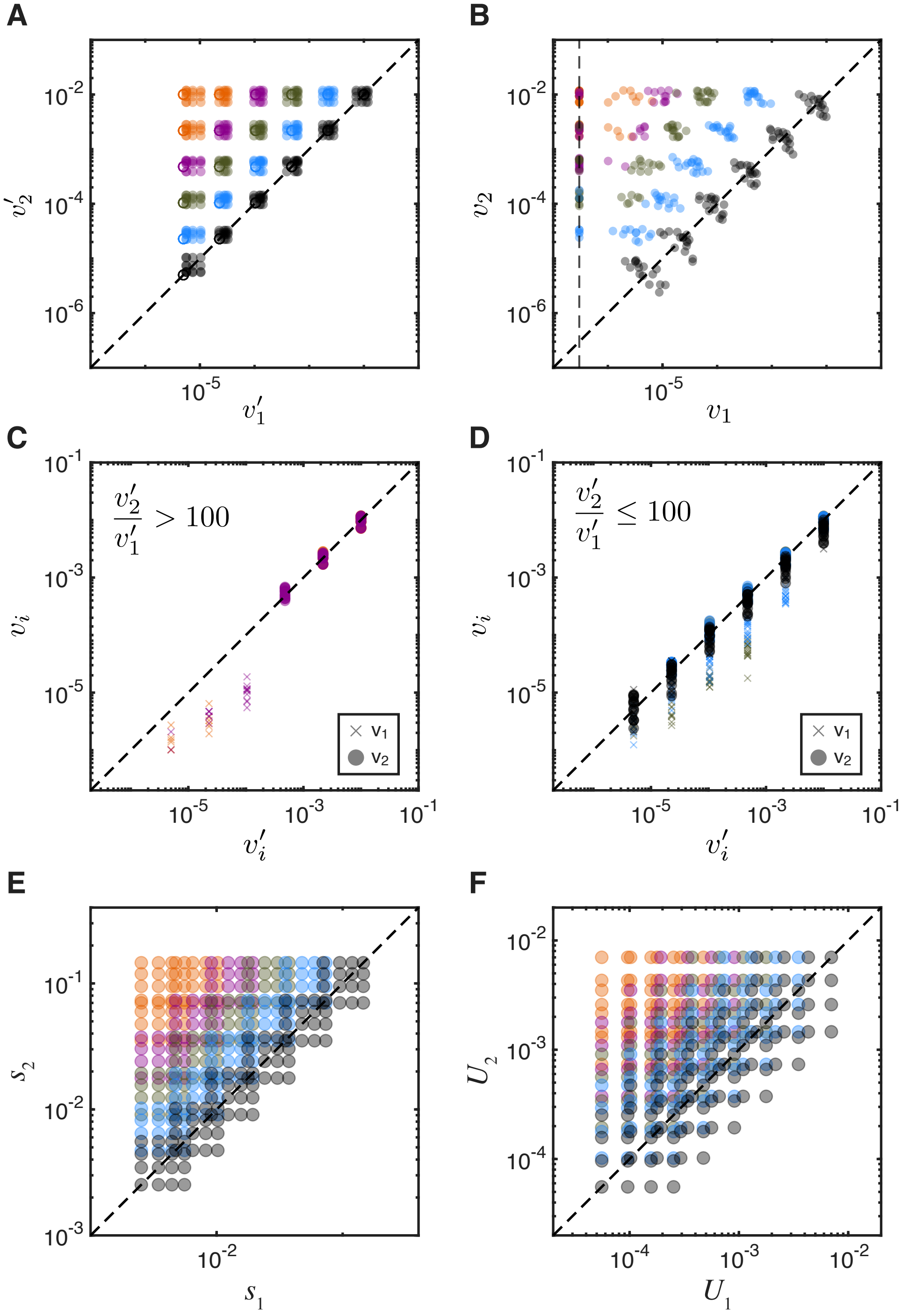
A
B
C
D
E
F
$\frac{v_2'}{v_1'} > 100$
$\frac{v_2'}{v_1'} \leq 100$
v_2'
v_1'
v_2
v_1
v_i
v_i'
s_2
s_1
U_2
U_1
v1
v2

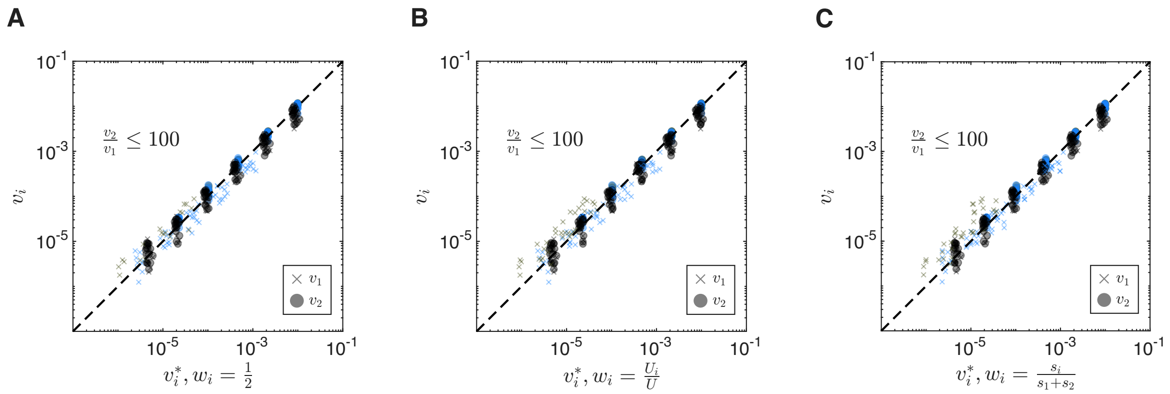

Figure S2. Accuracy of the heuristic approximation for various choices of weights w_i. We plot the empirically estimated rates of adaptation in a two-module organism v_i against those predicted theoretically using equation (S9) in the simultaneous adaptation regime ($v_2'/v_1' \leq 100$). Crosses ($\times$) denote v_1; filled circles denote v_2. Colors correspond to Figure S1A. Each panel corresponds to a different choice of weights w_i for the calculation of the effective selection coefficient $\tilde{s}$ (equation (S7)), as indicated. The dashed line represents $v_i = v_i^*$ in all panels.

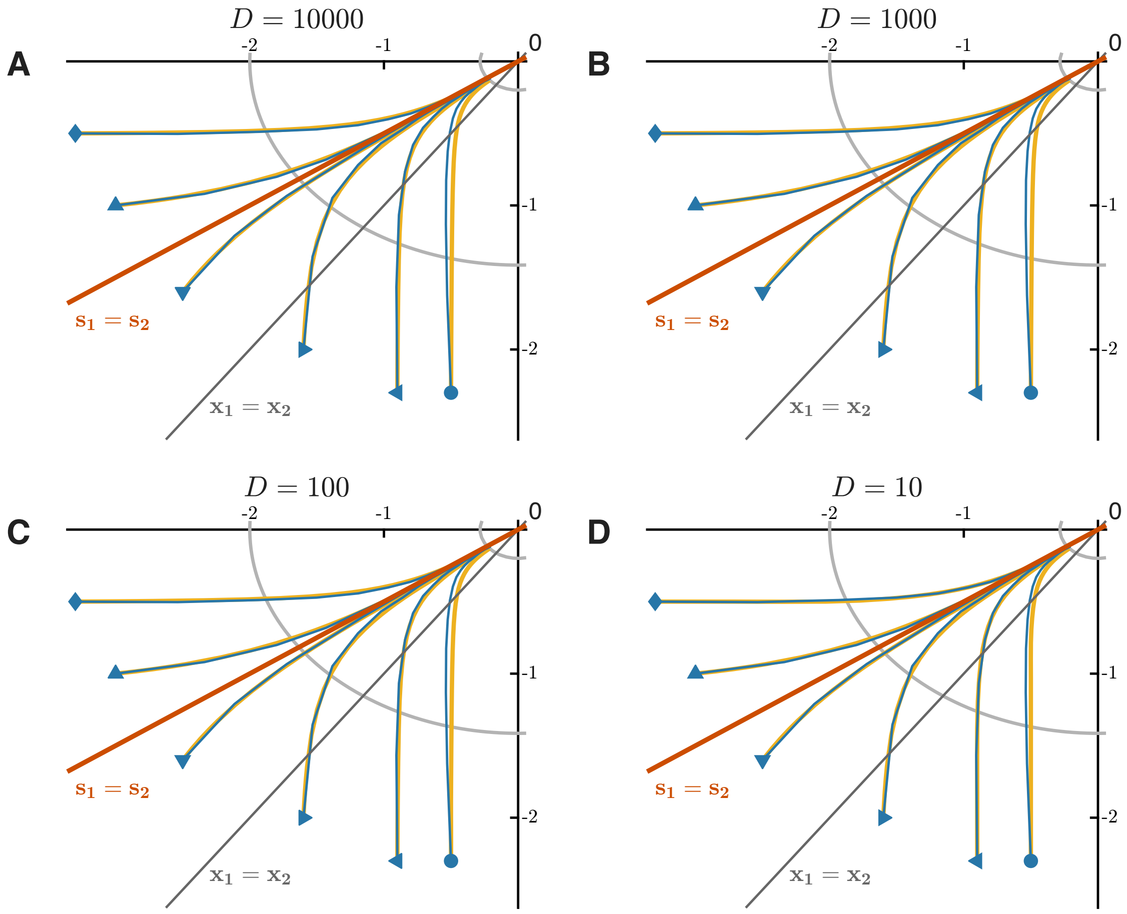

Figure S3. Sensitivity analysis of the heuristic approximation with respect to the threshold parameter D**.** Each panel shows the same simulated mean trajectories as Figure 3B (concurrent mutations, complete linkage; see main text for simulation details), alongside theoretical predictions (yellow) computed with a different value of D, as indicated.

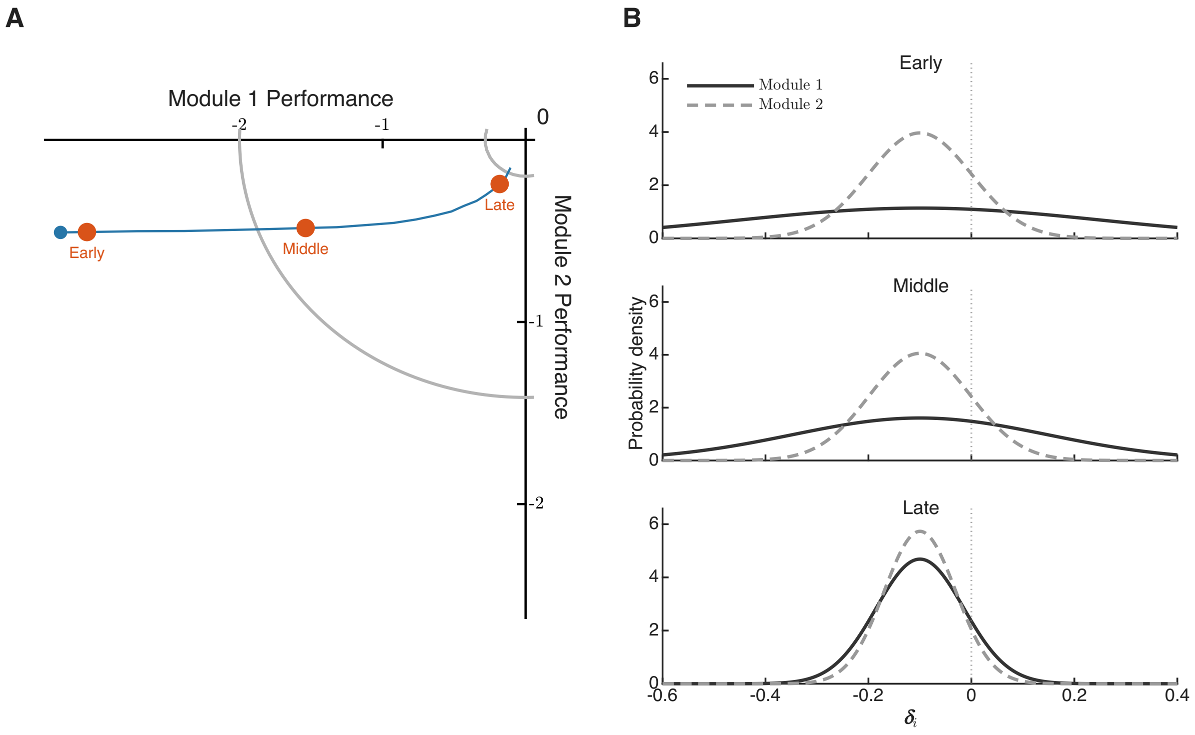

Figure S4. Mutation-effect distributions in the nested Fisher's Geometric Model change along an adaptive path. A. An example evolutionary trajectory in the trait space. Orange circles mark three points along the trajectory for which the distributions of phenotypic effects are shown in panel B. **B.** Distribution of phenotypic effects for module 1 (solid) and module 2 (dashed) at the three points in the trait space marked in panel A.

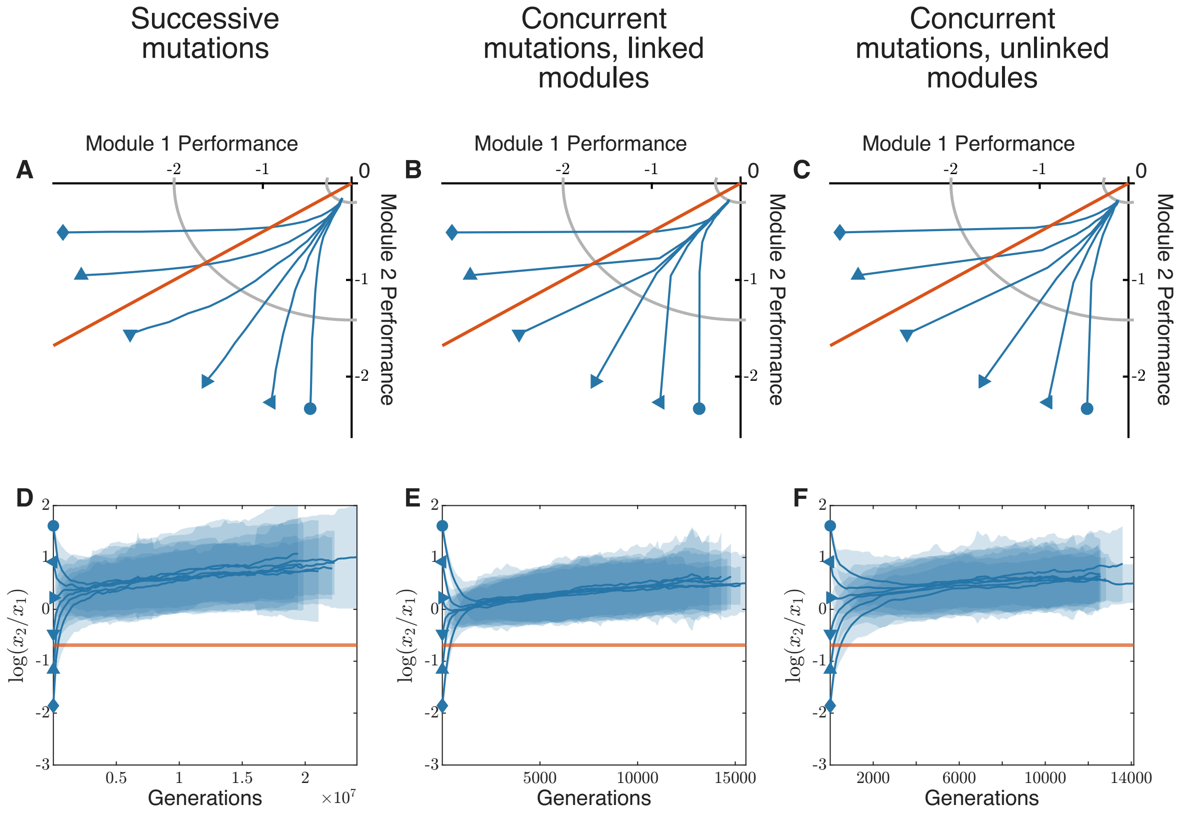

Figure S5. Evolutionary dynamics in the nested FGM model with different module dimensionalities. Same as Figure 5 in the main text, but with $n_1 = 10$, $n_2 = 20$.

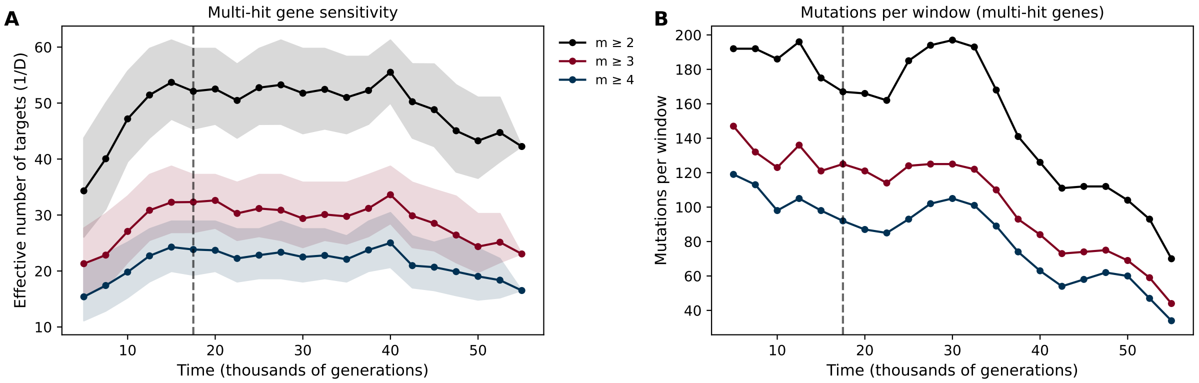

Figure S6. Sensitivity of the sliding-window analysis to the multiplicity threshold. A. Same as Figure 6 in the main text, but with mutations restricted to multi-hit genes with multiplicites m greater or equal to 2 (black; 271 mutations total, 71 subsampled), 3 (burgundy; 121 mutations total, 44 subsampled) or 4 (dark blue; 61 mutations total, 21 subsampled), as indicated in the legend. Slight declines at later time points are correlated with the overall decline in detections of mutations in multi-hit genes shown in panel B. **B.** Number of detected mutations per sliding window for each multiplicity threshold. Colors are the same as in panel A.
